# Shuxuetong injection for ischemic stroke: an overview of systematic reviews using different methodological quality assessment tools

**DOI:** 10.3389/fphar.2026.1723582

**Published:** 2026-04-13

**Authors:** Yifan Zhu, Xinxing Lai, Ying Gao, Yilan Zheng, Meilin Miao, Lu Tang

**Affiliations:** 1 Dongzhimen Hospital, Beijing University of Chinese Medicine, Beijing, China; 2 Beijing University of Chinese Medicine, Beijing, China; 3 Institute for Brain Disorders, Beijing University of Chinese Medicine, Beijing, China; 4 Dongfang Hospital, Beijing University of Chinese Medicine, Beijing, China

**Keywords:** certainty of evidence, ischemic stroke, methodological assessment, overview, Shuxuetong injection, systematic reviews

## Abstract

**Background:**

Shuxuetong injection possesses blood-activating and stasis-resolving effects with meridian-dredging functions. A growing body of evidence on Shuxuetong injection for the treatment of ischemic stroke has been published; however, no study has comprehensively assessed the methodological quality or clinical conclusions of that evidence. We conducted an overview of systematic reviews (SRs) on Shuxuetong injection for ischemic stroke to synthesize and critically assess the data, conclusions, and methodological quality of the included SRs, aiming to provide reliable evidence-based recommendations for clinical decision-making.

**Methods:**

Following a pre-registered protocol on PROSPERO, we conducted systematic searches across eight Chinese and English databases from inception to 25 January 2025. Two reviewers independently screened the SRs according to the eligibility criteria, and any disagreements were resolved through discussion to reach a consensus. Data were extracted for qualitative synthesis. The overlap in primary studies was quantitatively assessed. Methodological quality, reporting completeness, risk of bias, and certainty of evidence were evaluated using AMSTAR-2, PRISMA 2020, ROBIS, and GRADE tools.

**Results:**

This overview included 16 SRs. The overlap in primary studies was minimal. Likewise, subgroup analyses comparing conventional therapy (CT) versus Chinese medicine injection combined with CT also indicated low overlap. Methodological evaluation using AMSTAR-2 showed that all included reviews had critically low confidence. Based on PRISMA 2020 (score range: 24.5–31), 13 reviews were graded as meeting moderate-quality criteria and 3 as low quality, with no review rated as high quality. ROBIS assessment revealed a high risk of bias across all included reviews. GRADE evaluations of 125 outcomes showed moderate certainty for 7, low certainty for 21, and very low certainty for 97.

**Conclusion:**

Shuxuetong injection demonstrated variable efficacy in improving neurological deficits and activities of daily living, as well as in normalizing hemorheological and other laboratory parameters, with no significant increase in adverse events, indicating a favorable safety profile. However, the credibility of these findings is undermined by the predominantly low or very low quality of the included SRs, as assessed by methodological quality assessment tools. Our findings suggest that the design of future reviews should be rigorously improved in accordance with established methodological criteria to increase confidence in the conclusions.

**Systematic Review Registration:**

https://www.crd.york.ac.uk/PROSPERO/view/CRD42025638286, identifier CRD42025638286.

## Graphical Abstract

**Figure:**
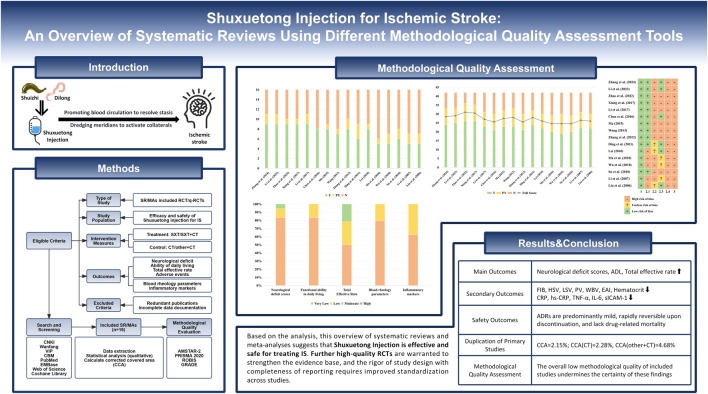

## 1 Introduction

Ischemic stroke (IS), also known as cerebral infarction, involves localized brain tissue ischemic necrosis or softening caused by obstruction of cerebral blood flow, leading to ischemia and hypoxia (Liu et al., 2023). Globally, IS accounts for approximately 70%–80% of new stroke cases, making it the most prevalent and serious manifestation of cerebrovascular events; consequently, it is the primary reason for hospitalization among patients with neurological disorders, particularly those with stroke-related conditions. IS ranks as the second leading global cause of mortality and the third predominant contributor to disability among neurological disorders (Feske, 2021; Feigin et al., 2021). This disease burden has been increasing socioeconomic costs worldwide, particularly in developing nations where incident rates show a persistent upward trajectory (Campbell et al., 2019; Feigin et al., 2022). Notably, regions with medium-to-high sociodemographic index (SDI) values demonstrate the highest global burden of IS, reflected in increased incidence, prevalence, mortality, and disability-adjusted life years (DALYs) (Li et al., 2024). Despite a decrease in global age-standardized incidence of IS, China has experienced a counter-trend increase of 34.7% in incident cases from 1990 to 2019 (Feigin et al., 2021; Ma et al., 2021). The treatment of regions with medium-to-high SDI scores bears a disproportionately heavy stroke-related burden, primarily attributable to suboptimal management of modifiable risk factors. The 2024 AHA/ASA primary stroke prevention guidelines prioritize eight modifiable risks: hypertension, dyslipidemia, diabetes/impaired glucose tolerance, obesity, sleep-disordered breathing, tobacco use, unhealthy diet, and physical inactivity (Bushnell et al., 2024). Consequently, refining IS interventions, while standardizing secondary prevention strategies, remains critical to mitigate disease burden.

Current clinical management pursues dual objectives: rapid restoration of cerebral perfusion within evidence-based time windows, and attenuation of secondary neuronal injury through neuroprotection targeting excitotoxicity and inflammation. Salvaging the ischemic penumbra serves as the cornerstone of these efforts (Herpich and Rincon, 2020). Therefore, intravenous thrombolysis (IVT) and endovascular thrombectomy (EVT) constitute first-line interventions for IS, owing to their proven efficacy in salvaging penumbra. However, a cross-sectional study using data from Bigdata Observatory Platform for Stroke of China (BOSC) revealed a median onset-to-door time of 24 h among 78,389 acute ischemic stroke (AIS) patients in 2020, with merely 11.79% presenting within the critical 3-h golden window (Yuan et al., 2023).

Treatment conditions and inter-patient variability constrain the clinical application of current interventions. To develop novel adjunctive therapies, TCM demonstrates favorable clinical outcomes despite incompletely elucidated multi-constituent mechanisms, highlighting its unique therapeutic value. Consequently, integrative TCM biomedicine approaches for IS management constitute an established clinical consensus, while the evidence base requires rigorous validation through multicenter trials. In TCM, IS is categorized as Zhongfeng, differentiated into the meridian-collateral pattern and the zangfu-organ pattern. The disease locus involves the brain and marrow sea, with pathological changes in the blood vessels. Its nature manifests as root deficiency with superficial excess. The acute phase predominantly presents excess patterns characterized by wind, fire, phlegm, and stasis; convalescent/chronic phases show deficiency patterns including qi–yin deficiency, phlegm–stasis, and blood stasis. Crucially, the blood stasis pattern constitutes the pathological cornerstone during IS progression, primarily dictating clinical outcomes (Liu and Gao, 2012). The blood stasis pattern is characterized by laboratory indicators, including vascular occlusion, hemorheological abnormalities, and coagulation dysfunction. Diseases with microcirculatory disturbance pathogenesis align with blood stasis pattern criteria. Blood-activating and stasis-resolving TCM therapies demonstrate efficacy in improving microcirculation, promoting angiogenesis, and exerting antithrombotic/antiplatelet effects, as substantiated by clinical studies (Gao et al., 2018). Given their rapid onset, multi-target effects, and precise targeting of ischemic stroke’s core pathology, Chinese medicine injections have become the most widely used adjunctive class in clinical practice. When combined with conventional therapy, they significantly improve neurological outcomes, reduce adverse events, and enhance long-term prognosis.

Shuxuetong injection, a Chinese medicine injection derived from Dilong (*Amynthas aspergillus* Perrier [Megascolecidae]) and Shuizhi (*Whitmania pigra* Whitman [Hirudinidae]), exhibits blood-activating and stasis-resolving properties with meridian-dredging effects. It is indicated for the acute phase of IS attributable to blood-stasis obstructing collaterals. The injection contains oligopeptides, free monosaccharides, oligosaccharides, amino acids, inorganic salts, and endogenous small molecules, with oligopeptide monomers demonstrating potent anticoagulant, fibrinolytic, and antiplatelet activities (Tang et al., 2024). Hirudin, the principal bioactive constituent of Shuizhi, acts as the most specific irreversible thrombin inhibitor by binding to thrombin’s exosite I and catalytic site, thereby suppressing fibrin formation and platelet activation (Matheson and Goa, 2000; Nowak, 2002; Junren et al., 2021). Lumbrokinase from Dilong mitigates cerebral ischemia-reperfusion injury by inhibiting the IRE1–XBP1 signaling pathway, reducing neuronal apoptosis and neuroinflammation (Wang et al., 2022). As a frequently prescribed Chinese medicine injection, its efficacy in improving neurological recovery and reducing hemorrhagic transformation has been validated by RCTs.

Accumulating evidence on Shuxuetong injection for IS has been published, evaluating its efficacy as an adjunct to thrombolysis, conventional antithrombotics, and specific subtypes, including stroke-in-progression (SIP). However, no study has comprehensively assessed the methodological rigor or clinical conclusions of this evidence. This overview critically appraises evidence from existing systematic reviews/meta-analyses, synthesizes data on efficacy/safety outcomes, and evaluates methodological quality, thereby generating evidence-based guidance for clinical decision making.

## 2 Methods

### 2.1 Project registration

The methodology of this review was registered with PROSPERO (Registration ID: CRD42025638286) prior to commencing formal screening, as stated in the predesigned protocol.

### 2.2 Inclusion and exclusion criteria

#### 2.2.1 Type of study

This systematic review encompasses randomized controlled trials (RCTs), including quasi-randomized controlled trials (q-RCTs). RCTs are regarded as the gold standard for evaluating therapeutic interventions and constitute the highest tier of evidence in evidence-based medicine, achieved through rigorous allocation concealment, blinding, and controlled comparison groups.

#### 2.2.2 Study population

This overview critically synthesizes evidence from systematic reviews and meta-analyses (SRs/MAs) that exclusively included RCTs or q-RCTs, with a focus on the efficacy and safety of Shuxuetong injection (SXT) for IS. The diagnosis of ischemic stroke required meeting at least one criterion: (1) adherence to the 1995 Diagnostic Criteria for Cerebral Infarction (China’s fourth National Conference on Cerebrovascular Diseases), (2) compliance with internationally recognized guidelines, or (3) imaging confirmation via cranial CT/MRI when formal criteria were unavailable.

#### 2.2.3 Interventions

Our study utilizes Shuxuetong injection (Chinese National Drug Approval No. Z20010100), manufactured by Mudanjiang Youbo Pharmaceutical Co., Ltd., which complies with the Chinese National Drug Standard WS3-548 (Z-084)-2005(Z). The formulation contains Dilong (*Amynthas aspergillus* Perrier [Megascolecidae]) and Shuizhi (*Whitmania pigra* Whitman [Hirudinidae]) (Table 1), with a standard dosage of 6 mL. The manufacturing process involves initial steps such as washing, soaking, and grinding of the raw materials, which are also subjected to X-ray inspection for quality control. The extraction of active components is subsequently carried out using the freeze–thaw method, a technique that induces cell lysis through cyclic freezing and thawing to increase compound yield. The injection contains a variety of chemical constituents, including amino acids, oligosaccharides, organic acids and bases, short-chain fatty acids, and small peptides (Tang et al., 2024).

**TABLE 1:** Species of medicinal materials in Shuxuetong Injection.

Classification	Dilong	Shuizhi
Kingdom	Animalia	Animalia
Phylum	Annelida	Annelida
Class	Clitellata	Clitellata
Subclass	Oligochaeta Grube, 1850	-
Order	Crassiclitellata Jamieson, 1988	Arhynchobdellida
Family	Megascolecidae Rosa, 1891	Hirudinidae Whitman, 1886
Genus	*Amynthas* Kinberg, 1866	*Whitmania* Blanchard, 1888
Species	*Amynthas aspergillus* (Perrier, 1872)	*Whitmania pigra* (Whitman, 1884)

Interventions were assigned as follows.

Trial group: (a) SXT, monotherapy; or (b) SXT, combined with conventional therapy (CT).

Control group: (a) CT, alone; (b) CT, combined with active comparators (e.g., other Chinese medicine injections); or (c) CT, combined with inert placebo.

#### 2.2.4 Outcomes

Outcomes were predefined as primary or secondary in the study protocol.

Primary outcomes included changes in neurological deficit assessed by the National Institutes of Health Stroke Scale (NIHSS) or the Chinese Stroke Scale (CSS), improvements in daily living ability measured via the Barthel index, and total response rate. Safety outcomes encompassed the incidence of adverse events. Secondary outcomes comprised blood rheology parameters, including fibrinogen (FIB), whole-blood high-shear viscosity (HSV), whole-blood low-shear viscosity (LSV), plasma viscosity (PV), etc.

#### 2.2.5 Exclusion criteria

Redundant publications and literature with incomplete data documentation.

### 2.3 Search strategy

We employed a combined subject heading and free-text search strategy across eight databases: China National Knowledge Infrastructure (CNKI), Wanfang Data, VIP Information Database, Chinese Biomedical Literature Database (CBM), PubMed, Embase, Web of Science, and Cochrane Library. Search terms comprised ischemic stroke, cerebral infarction, systematic review, meta-analysis, and Shuxuetong injection. We manually screened the reference lists of all included studies. The search period extended from database inception to 25 January 2025 (Supplementary Appendix 1).

### 2.4 Literature screening and data extraction

Duplicate records were first removed using NoteExpress software, followed by manual inspection to identify residual duplicates. Two researchers independently screened titles and abstracts using predefined eligibility criteria. Full texts of potentially eligible studies were then retrieved and independently reviewed, with disagreements resolved by consensus. Disagreements were resolved through consensus or third-party adjudication. Final inclusion decisions were documented with reasons for exclusion.

During the data extraction phase, two independent researchers utilized a Microsoft Excel 2019 template to systematically extract essential information. Discrepancies were resolved through consensus or third-party arbitration. The standardized form captured: (1) study identification (first author, publication year); (2) type and number of original studies and total sample size; (3) intervention parameters specifying experimental therapy versus comparator regimen; (4) treatment duration; (5) primary/secondary outcomes with measurement scales; and (6) meta-analysis results reporting pooled effect size.

### 2.5 Statistical methods

To evaluate duplication of primary studies across multiple systematic reviews, we employed a citation matrix approach to quantify original study overlap, calculating the corrected covered area (CCA) using the following formula (Cheng et al., 2023):

$$CCA=\frac{N-r}{\left(r\times c\right)-r},$$

where N = total primary studies included duplicates; r = number of RCTs; c = number of SRs.

Based on thresholds established by Pieper et al. (2014), a CCA value below 5% signifies minimal overlap with a low risk of bias, values between 5% and 15% indicate moderate overlap requiring cautious interpretation of results, and values exceeding 15% reflect substantial overlap with a high risk of bias, necessitating sensitivity analysis. In instances where CCA exceeded 15%, we performed sensitivity analyses by excluding duplicate studies; if bias persisted, we repeated the meta-analyses using deduplicated datasets to ensure robustness.

### 2.6 Methodological quality assessment

Methodological quality of included SRs/MAs was appraised using AMSTAR-2, with item-level ratings summarized as frequencies. Reporting completeness was evaluated against PRISMA 2020, documenting adherence rates per checklist item. Risk of bias was assessed using ROBIS, which categorizes judgments (low/high/unclear) across its three phases. Evidence certainty for outcomes was graded using GRADE, quantifying downgrading reasons across five domains. Results were structured into tables in Microsoft Excel 2019.

## 3 Results

### 3.1 Literature screening

**FIGURE 1:**
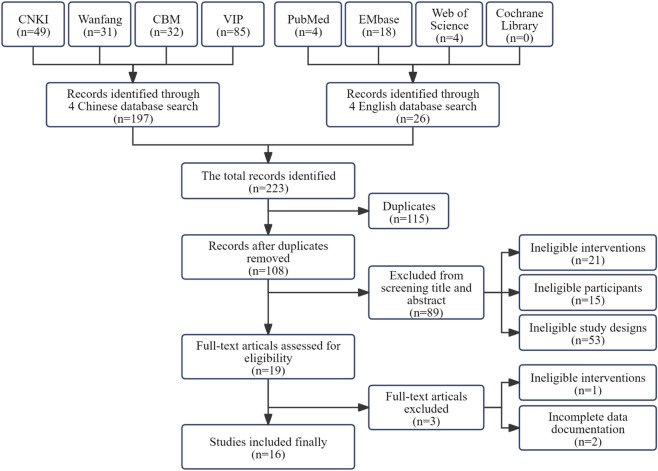
Flow diagram of study selection.

A systematic search across eight databases (CNKI, Wanfang Data, VIP Chinese Journal Database, SinoMed/CBM, PubMed, Embase, Web of Science, and Cochrane Library) identified 223 records. After removing 115 duplicates using NoteExpress reference management software and excluding 89 records through title/abstract screening, 19 articles underwent full-text assessment. Three studies were excluded due to ineligible interventions (n = 1) and insufficient outcome data (n = 2). Sixteen studies were included in the final qualitative synthesis (Figure 1).

### 3.2 Literature characteristics

Sixteen Chinese-language SRs/MAs met the inclusion criteria (2006–2024) (Zhang et al., 2024; Li et al., 2023; Zhao et al., 2022; Xiang et al., 2017; Li et al., 2017; Chen et al., 2016; Ma, 2015; Wang, 2013; Zhang et al., 2012; Ding et al., 2011; Lei, 2010; Ma and Li, 2010; Wu et al., 2010; Su et al., 2010; Li and Miao, 2007; Liu et al., 2006), comprising 13 journal articles and 3 theses. Eleven focused on AIS, three on IS, and two on SIP. Diagnostic criteria were explicitly stated in 13 studies; one used radiologically confirmed diagnoses (CT/MRI) without specified criteria, while two cited authoritative but unnamed standards (Ma and Li, 2010; Li and Miao, 2007). Symptom onset was restricted (e.g., ≤72 h for AIS) in 10 studies. Two studies included q-RCTs (Zhang et al., 2012; Lei, 2010); others included only RCTs. Each meta-analysis synthesized 4–31 primary studies (total samples: 393–2,150 participants). Treatment duration was 10–25 days (median 14–15 days). Interventions involved SXT + CT versus controls: CT alone (n = 7), other neuroprotective agents (n = 6), or alternative therapies (n = 2) (Table 2).

**TABLE 2:** Basic characteristics of the included SRs and MAs.

Included SRs/MAs (Years)	Type of primary studies	Number of primary studies (sample size)	Course of IS	Treatment duration	Intervention measures		Outcomes
					Treatment group	Control group	
Zhang et al. (2024)	RCT	8 (535)	<4.5 h	14 d	SXT + CT	CT	①②③④⑥⑦
Li et al. (2023)	RCT	31 (1142)	>24 h	7-30 d	SXT + CT	CT	①②③④⑥⑦
Zhao et al. (2022)	RCT	22 (2090)	>7 d	10-28 d	SXT + CT	CT	①②③④⑤⑥⑦⑧
Xiang et al. (2017)	RCT	8 (822)	Unclear	14-15 d	SXT + CT	CT	①②③④⑤⑥
Li et al. (2017)	RCT	11 (701)	<72 h	12-21 d	SXT + CT	*Ginkgo biloba* extract injection + CT	①②③④
Chen et al. (2016)	RCT	13 (1241)	<72 h	14-28 d	SXT + CT	Notoginseng injection + CT	①②③④
Ma (2015)	RCT	15 (1457)	<72 h	10-21 d	SXT + CT	Xuesaitong injection + CT	①③④⑥
Wang (2013)	RCT	18 (1962)	Unclear	10-20 d	SXT + CT	Other injection + CT	①③④⑥
Zhang et al. (2012)	RCT/q-RCT	11 (972)	<72 h	14-30 d	SXT + CT	CT	①③④⑤
Ding et al. (2011)	RCT	10 (938)	Unclear	10-15 d	SXT + CT	Other injection + CT	①③④
Lei (2010)	RCT/q-RCT	13 (1296)	<14 d	14-20 d	SXT + CT	CT/other injection + CT	①③⑥
Ma and Li (2010)	RCT	24 (2150)	<30 d	5-21 d	SXT + CT	CT	③
Wu et al. (2010)	RCT	28 (1654)	Unclear	10-15 d	SXT + CT	CT/other injection + CT	①③④
Su et al. (2010)	RCT	11 (967)	<72 h	14 d	SXT + CT	CT	③
Li and Miao (2007)	RCT	4 (393)	<30 d	14-21 d	SXT + CT	CT	①②③④
Liu et al. (2006)	RCT	11 (1122)	Unclear	Unclear	SXT + CT	Other injection + CT	③④⑥⑦⑧

① Neurological deficit score, ② competence in daily living score, ③ effective rate, ④ adverse events, ⑤ case fatality rate, ⑥ blood rheology parameters, ⑦ inflammatory markers, ⑧ coagulation parameters.

### 3.3 Main outcomes

#### 3.3.1 Neurological deficit scores

Fifteen SRs assessed neurological deficit outcomes using standardized scales.

Five studies used the NIHSS to evaluate improvement (Zhang et al., 2024; Li et al., 2023; Zhao et al., 2022; Xiang et al., 2017; Lei, 2010), all of which indicated that SXT + CT resulted in significantly greater NIHSS reductions than CT alone. Significant heterogeneity was observed in four studies, and one study conducted a subgroup analysis stratified by NIHSS assessment timepoints (Zhang et al., 2024), which showed that the SXT group exhibited no significant improvement in NIHSS scores at 24-h post-treatment. However, scores at 14 days were substantially lower than those recorded at the 24-h, 48-h, and 7-day timepoints. One investigation conducted a sensitivity analysis to address substantial heterogeneity (Zhao et al., 2022). After excluding an outlier from the original study enrolling patients with milder baseline stroke severity, the SXT group showed a clinically meaningful reduction in NIHSS scores compared to the CT group. In contrast, two studies provided no discussion on heterogeneity sources (Li et al., 2023; Xiang et al., 2017).

Ten reviews assessed neurological improvement using CSS in IS cohorts (Zhao et al., 2022; Xiang et al., 2017; Chen et al., 2016; Ma, 2015; Zhang et al., 2012; Ding et al., 2011; Ma and Li, 2010; Wu et al., 2010; Li and Miao, 2007; Liu et al., 2006). Significant heterogeneity was identified in six reviews. Subgroup analysis stratified by control interventions demonstrated significantly greater CSS score reductions with SXT versus compound Danshen injection and puerarin, while showing comparable efficacy to low-molecular-weight heparin (Ding et al., 2011). Sensitivity analyses in three reviews confirmed consistency with primary outcomes (Ma, 2015; Ma and Li, 2010; Li and Miao, 2007), supporting result robustness. Potential heterogeneity sources remained unexplored in two reports (Xiang et al., 2017; Chen et al., 2016). Among the included reviews, one conducted subgroup analyses by comparator drug (Safflower injection, low-molecular-weight heparin calcium, and troxerutin) (Liu et al., 2006). The analysis demonstrated that SXT significantly reduced CSS scores compared to Safflower injection but had comparable efficacy to both low-molecular-weight heparin calcium and troxerutin. Another review did not perform subgroup analyses by control drug (Wu et al., 2010); its overall analysis showed no significant heterogeneity and a statistically significant CSS score reduction with SXT versus controls.

Three SRs did not conduct subgroup analyses to explore heterogeneity related to different neurological impairment scales (Li et al., 2017; Wang, 2013; Lei, 2010).

#### 3.3.2 Functional ability in daily living score

Functional ability in daily living was assessed using standardized measures, including the Barthel index (BI), across six SRs (Zhang et al., 2024; Li et al., 2023; Zhao et al., 2022; Li et al., 2017; Chen et al., 2016; Li and Miao, 2007). Among these, two SRs addressed high baseline heterogeneity through sensitivity analyses; after sequentially excluding one outlier study substantially contributing to heterogeneity, both achieved resolved heterogeneity and demonstrated statistically significant improvements in BI scores favoring the intervention group versus controls (Zhang et al., 2024; Zhao et al., 2022). Separately, one review comparing the intervention to *Ginkgo biloba* extract injection reported no significant heterogeneity with statistically superior BI outcomes in the intervention group, confirming consistent therapeutic benefits (Li et al., 2017).

#### 3.3.3 Total effective rate

Total effective rate was assessed using historical criteria (1995) from the Chinese Medical Association, including the neurological deficit scale (CSS) or the National Institutes of Health Stroke Scale (NIHSS). Efficacy tiers were defined as follows: score reductions of 91%–100% denoted excellent improvement, 46%–90% denoted marked improvement, 18%–45% denoted moderate improvement, and 0%–17% denoted no change/ineffective, with score increases or death classified as deterioration. Consequently, the total response rate was calculated as the proportion of patients achieving ≥18% score reduction (encompassing the excellent/marked/moderate improvement categories).

Thirteen SRs used the total effective rate as an outcome measure (Zhang et al., 2024; Li et al., 2023; Zhao et al., 2022; Xiang et al., 2017; Li et al., 2017; Chen et al., 2016; Ma, 2015; Wang, 2013; Zhang et al., 2012; Ding et al., 2011; Ma and Li, 2010; Li and Miao, 2007; Liu et al., 2006).

1. SXT + CT versus CT alone

Seven SRs compared SXT + CT versus CT alone. All demonstrated homogeneous results and significantly superior clinical efficacy (Zhang et al., 2024; Li et al., 2023; Zhao et al., 2022; Xiang et al., 2017; Zhang et al., 2012; Ma and Li, 2010; Li and Miao, 2007).

In one review, the primary studies were stratified by neurological assessment scale (NIHSS or CSS) and SXT dosage (6 mL/day, 8 mL/day, or 10 mL/day), with all experimental groups receiving clopidogrel 75 mg/day. Subgroup analyses consistently showed minimal heterogeneity and statistically significant efficacy improvements, supporting the robustness of the conclusion (Xiang et al., 2017).

2. SXT + CT versus other active comparator injections + CT

The category of pharmacotherapies includes Chinese medicine injections, such as *Ginkgo biloba* extract injection, Shuxuening injection, and compound Danshen injection. It also encompasses conventional biomedicine agents like troxerutin and low-molecular-weight heparin (LMWH). Their primary pharmacological mechanisms involve anticoagulation, antiplatelet aggregation, vasodilation, improvement of microcirculation, and anti-inflammatory effects, collectively contributing to antithrombotic outcomes. These agents are frequently employed in the clinical management of IS.

Six SRs compared SXT + CT versus other active comparator injections + CT (Li et al., 2017; Chen et al., 2016; Ma, 2015; Wang, 2013; Ding et al., 2011; Liu et al., 2006).

Subgroup analyses across three SRs evaluated differential efficacy against specific control drugs: one compared interventions with *Ginkgo biloba* extract (including Ginkgo Damo injection and Shuxuening injection) (Li et al., 2017); one with compound Danshen injection (Wang, 2013); two with ligustrazine injection (Wang, 2013; Liu et al., 2006); one with Xuesaitong injection (Wang, 2013); one with Xueshuantong injection (Wang, 2013); two with Safflower injection (Wang, 2013; Liu et al., 2006); and one with Danshen Injection (Liu et al., 2006). All subgroup comparisons demonstrated statistically significant differences. One review utilized Xuesaitong injection as the intervention in the control group, and the difference also demonstrated statistical significance (Ma, 2015). One reported comparable total response rate between SXT and low-molecular-weight heparin (LMWH) or puerarin injections in IS (Wang, 2013). Similarly, another demonstrated equivalent outcomes for SXT versus Notoginseng, Xuesaitong, LMWH calcium, troxerutin, and Xueshuantong Injections (Liu et al., 2006). Two reviews showed statistically significant results without heterogeneity despite lacking subgroup analyses for specific controls (Chen et al., 2016; Ding et al., 2011).

#### 3.3.4 Adverse events and safety

A total of 12 SRs reported adverse events (AEs) (Zhang et al., 2024; Li et al., 2023; Zhao et al., 2022; Xiang et al., 2017; Li et al., 2017; Chen et al., 2016; Ma, 2015; Wang, 2013; Lei, 2010; Ma and Li, 2010; Li and Miao, 2007; Liu et al., 2006).

Two reviews described serious AEs (SAEs): one documented gastrointestinal bleeding (n = 3), mucosal bleeding (n = 2), symptomatic intracranial hemorrhage (n = 7), nausea (n = 13), and vomiting (n = 12) (Zhang et al., 2024); another reported dizziness (n = 3), palpitations (n = 4), chest tightness (n = 1), and facial flushing with cephalic distension (n = 2) (Zhao et al., 2022).

The remaining 10 SRs noted non-serious AEs: subcutaneous ecchymosis (n = 4) (Xiang et al., 2017; Lei, 2010; Ma and Li, 2010; Li and Miao, 2007); mild gastrointestinal discomfort (n = 2) (Xiang et al., 2017); mild gastrointestinal discomfort, dizziness, and chest tightness resolved spontaneously after discontinuation of medication (Li et al., 2017); rash (n = 2) (Ma, 2015); rash (n = 2) with pruritus (n = 1), transient fibrinogen reduction, and petechiae (Wang, 2013); low-grade fever resolving spontaneously within days (n = 2) (Liu et al., 2006). One review focusing on mild allergic reactions conducted a meta-analysis showing no statistically significant increase in risk (Chen et al., 2016). Another omitted detailed reporting of non-serious AEs (Li et al., 2023).

### 3.4 Secondary outcomes

#### 3.4.1 Fibrinogen

A total of eight SRs reported fibrinogen outcomes, all demonstrating statistically significant differences (Zhang et al., 2024; Li et al., 2023; Zhao et al., 2022; Xiang et al., 2017; Ma, 2015; Wang, 2013; Lei, 2010; Liu et al., 2006). Among these, four SRs used CT as the comparator (Zhang et al., 2024; Li et al., 2023; Zhao et al., 2022; Xiang et al., 2017), while three employed other Chinese medicine injections (Ma, 2015; Wang, 2013; Lei, 2010). Of the latter, one specifically compared the intervention with Xuesaitong (Ma, 2015); the other two did not conduct subgroup analyses across distinct Chinese medicine comparators. Notably, one review utilized *Panax notoginseng* saponin injection as the control, including only one RCT (Liu et al., 2006).

Substantial heterogeneity was observed across all eight SRs. Only one review performed a sensitivity analysis to address this issue, revealing no qualitative change in clinical or statistical significance versus the original outcome, indicating result robustness (Zhao et al., 2022). The other seven SRs omitted a detailed exploration of heterogeneity sources.

#### 3.4.2 Whole-blood high-shear viscosity

Six SRs analyzed HSV (Zhang et al., 2024; Xiang et al., 2017; Ma, 2015; Wang, 2013; Lei, 2010; Liu et al., 2006). Significant differences were observed in five SRs, whereas one review demonstrated non-significant outcomes. The discrepancy is potentially attributable to restricted RCT enrollment (Ma, 2015). Among four studies exhibiting substantial heterogeneity (Zhang et al., 2024; Xiang et al., 2017; Wang, 2013; Lei, 2010), sensitivity analysis excluded an outlier, resolving variability while maintaining significance (Zhang et al., 2024). The remaining three SRs lacked detailed discussion on the sources of heterogeneity; notably, two of these employed distinct comparator drugs but did not conduct subgroup analyses to explore variations across different control interventions (Wang, 2013; Lei, 2010). One review exclusively incorporated a single RCT (Liu et al., 2006).

#### 3.4.3 Whole-blood low-shear viscosity

Six SRs reported outcomes on LSV (Zhang et al., 2024; Xiang et al., 2017; Ma, 2015; Wang, 2013; Lei, 2010; Liu et al., 2006). Of these, four exhibited substantial heterogeneity (Zhang et al., 2024; Xiang et al., 2017; Ma, 2015; Wang, 2013). One review addressed this issue through a sensitivity analysis that excluded an outlier that was the primary source of heterogeneity (Zhang et al., 2024). After exclusion, heterogeneity was resolved, and a statistically significant effect emerged. The remaining three SRs did not comprehensively investigate the sources of heterogeneity. One reported non-significant results, possibly attributable to low statistical power from the small number of included RCTs (n = 2; Ma, 2015). Two reviews employed alternative comparator drugs but omitted subgroup analyses for these variations (Wang, 2013; Lei, 2010); this omission may have confounded outcomes due to unadjusted confounding factors. One showed low heterogeneity (I^2^<25%; Wang, 2013; Lei, 2010), although this metric alone does not validate methodological quality. Finally, one review analyzed data from a single RCT, precluding meta-analytic synthesis (Liu et al., 2006).

#### 3.4.4 Plasma viscosity

Among six SRs analyzing PV outcomes (Zhang et al., 2024; Li et al., 2023; Xiang et al., 2017; Wang, 2013; Lei, 2010; Liu et al., 2006), four demonstrated considerable heterogeneity without detailed reviews into potential sources (Li et al., 2023; Xiang et al., 2017; Wang, 2013; Lei, 2010). The remaining two reviews have reported statistically non-significant results, potentially linked to inadequate statistical power stemming from restricted sample sizes (Zhang et al., 2024; Liu et al., 2006).

#### 3.4.5 Whole-blood viscosity

Three SRs reported statistically significant outcomes for WBV (Li et al., 2023; Xiang et al., 2017; Lei, 2010). Two exhibited substantial heterogeneity (I^2^>75%; Li et al., 2023; Xiang et al., 2017); both used CT as the control intervention, yet demonstrated significant WBV reduction versus baseline. The remaining study, showing low heterogeneity (I^2^<25%), compared SXT to other active comparators (compound Danshen injection and Xuesaitong injection) in two RCTs (Lei, 2010).

#### 3.4.6 Erythrocyte aggregation index

Three SRs reported outcomes for the erythrocyte aggregation index (EAI) (Xiang et al., 2017; Wang, 2013; Liu et al., 2006). In two reviews using other active comparators (compound Danshen injection and ligustrazine injection), pooled data from three RCTs (including one overlapping trial population) demonstrated significant EAI reduction (Wang, 2013; Liu et al., 2006). The remaining review reported non-significant findings, likely attributable to low statistical power from limited sample sizes (Xiang et al., 2017). Notably, substantial heterogeneity was observed in two reviews, yet potential sources were not investigated (Xiang et al., 2017; Wang, 2013).

#### 3.4.7 Hematocrit

Three SRs reported hematocrit outcomes comparing the intervention against other active comparators (Wang, 2013; Lei, 2010; Liu et al., 2006). One review demonstrated substantial heterogeneity (Lei, 2010). The remaining two reviews showed no statistically significant difference versus controls: one found that SXT did not differ significantly from comparators in reducing hematocrit levels (Wang, 2013), while another reported similar reductions between SXT and notoginsenoside Injection (Liu et al., 2006).

#### 3.4.8 Inflammatory markers

Four SRs assessed inflammatory markers, including high-sensitivity C-reactive protein (hs-CRP), C-reactive protein (CRP), tumor necrosis factor-α (TNF-α), interleukin-6 (IL-6), and soluble intercellular adhesion molecule-1 (sICAM-1) (Zhang et al., 2024; Li et al., 2023; Zhao et al., 2022; Liu et al., 2006). Control groups received CT in two reviews (Zhang et al., 2024; Li et al., 2023) and troxerutin injection in another (Liu et al., 2006). Hs-CRP was assessed in two reviews (both showing substantial heterogeneity) (Li et al., 2023; Zhao et al., 2022), CRP in one (Zhang et al., 2024), and TNF-α in three (one exhibiting significant heterogeneity; Zhang et al., 2024; Zhang et al., 2024; Li et al., 2023; Liu et al., 2006). IL-6 and sICAM-1 were each evaluated in one review (Zhao et al., 2022; Liu et al., 2006). All reported outcomes demonstrated statistically significant improvements versus controls.

### 3.5 Duplication of original studies

During the overview process, researchers identified substantial overlap in primary studies across multiple SRs and MAs. Although this study primarily employs qualitative synthesis, high overlap may overweight specific studies, inflating their statistical influence and potentially biasing conclusions. To quantify this bias, we constructed a citation matrix to document the inclusion frequency of each primary study.

This analysis included 16 systematic reviews, incorporating 180 unique primary studies with 238 total inclusions due to overlaps. The corrected covered area (CCA) was 2.15%, indicating slight overlap per Pieper’s classification and demonstrating minimal redundancy and high independence among the primary evidence bases.

Given heterogeneity in control group interventions, we stratified systematic reviews into two cohorts: CT alone versus other injections + CT. The CT cohort comprised eight systematic reviews, incorporating 100 unique primary studies with 119 total inclusions. CCA calculation (N = 119, r = 100, c = 8) gave 2.28%. The Chinese medicine injection plus CT cohort included eight SRs, encompassing 80 unique primary studies with 119 inclusions. CCA calculation (N = 119, r = 80, c = 8) gave 4.68%. Both CCA values indicate slight overlap (0%–5% per Pieper classification), confirming minimal redundancy among primary studies in each cohort (Supplementary Appendix Table 1).

### 3.6 Methodological quality assessment

#### 3.6.1 AMSTAR-2

**FIGURE 2:**
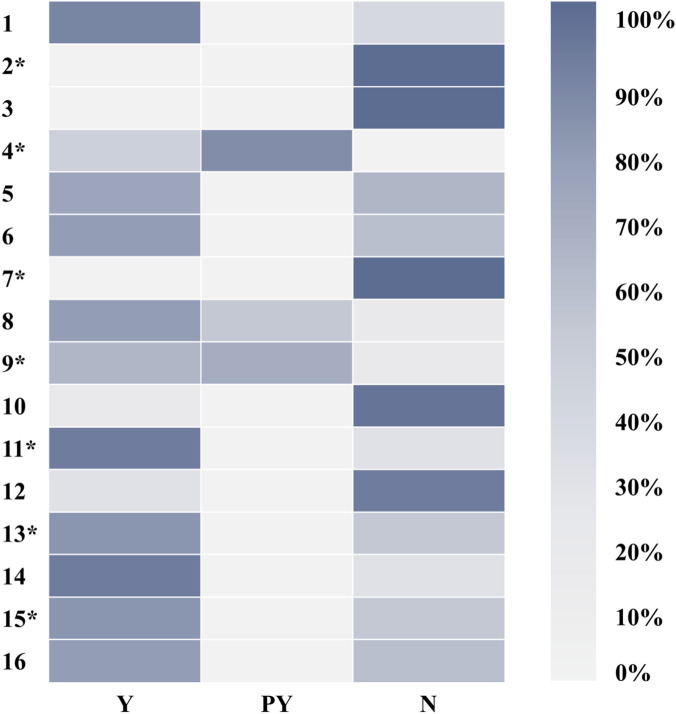
Methodological quality of included SRs assessed using the AMSTAR-2 tool (by review).

**FIGURE 3:**
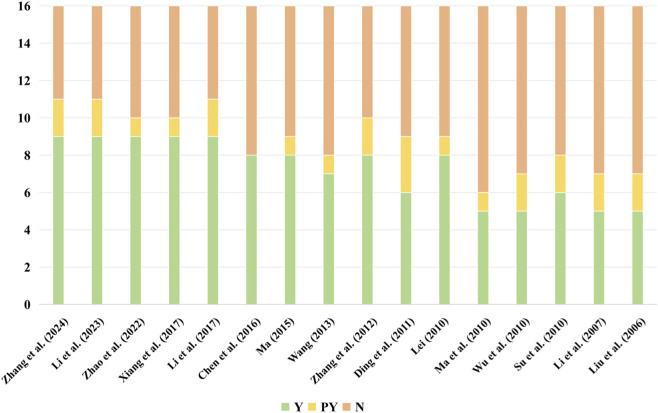
Methodological quality of included SRs assessed using the AMSTAR-2 tool (by item).

Researchers evaluated the methodological quality of the included studies using the AMSTAR-2 tool and its interpretation guidelines (Zhang et al., 2018). Because none of the included reviews provided a predefined protocol or a list of excluded studies with justification (failing Critical Items 2 and 7 of AMSTAR-2), all were rated as critically low confidence according to the tool’s criteria. Fewer than 50% of reviews met the criteria (fully or partially) for five AMSTAR-2 items (Items 2, 3, 7, 10, 12), indicating prevalent deficiencies in protocol registration, justification for study designs, documentation of excluded studies, reporting of funding sources in primary studies, and consideration of risk of bias in synthesizing results (Figure 2; Figure 3, Supplementary Appendix Table 2).

#### 3.6.2 PRISMA 2020

**FIGURE 4:**
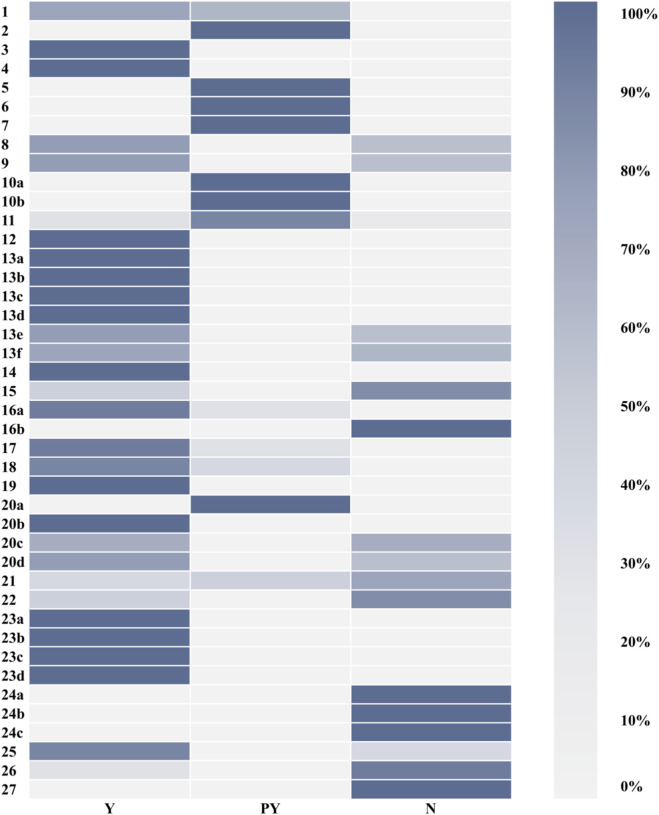
Quality of reporting of included SRs assessed using the PRISMA 2020 statement (by review).

**FIGURE 5:**
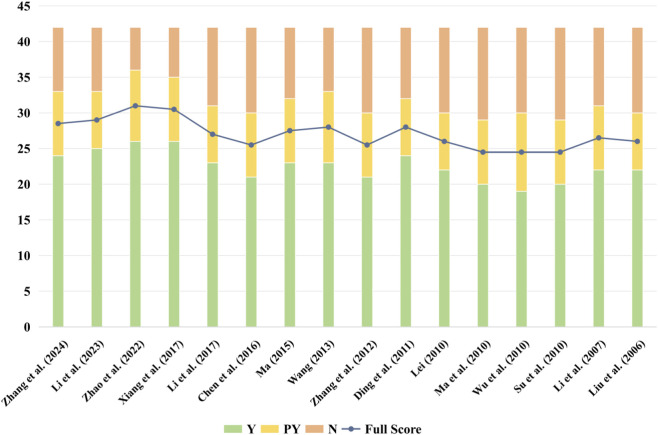
Quality of reporting of included SRs assessed using the PRISMA 2020 statement (by item).

During quality assessment of the included systematic reviews, researchers identified missing reporting items across all reviews. To standardize reporting completeness, they evaluated reviews using the PRISMA 2020 checklist and interpretation guidelines (Gao et al., 2021). Total scores ranged from 24.5 to 31 points, with 13 reviews rated moderate quality, 3 as low quality, and none as high quality. Seventeen items were fully reported in fewer than 50% of reviews. Five items (16b, 24a, 24b, 24c, and 27) were unreported in all reviews, indicating that no reviews provided: (1) justifications for excluding near-eligible studies, (2) protocol registration/declaration, or (3) public access to data/code/materials (Figure 4; Figure 5, Supplementary Appendix Table 3).

#### 3.6.3 ROBIS

**FIGURE 6:**
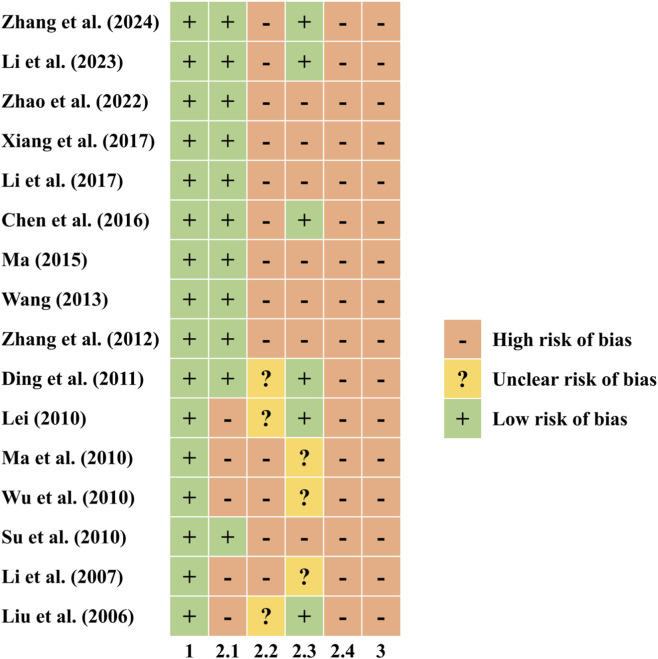
Risk of bias of included SRs with ROBIS tool.

During the quality assessment of the 16 included systematic reviews, researchers identified a high risk of bias across all reviews. To pinpoint specific bias sources, the ROBIS tool (developed in 2014 by Bristol University’s Department of Social Medicine for assessing bias in systematic reviews/meta-analyses to support clinical decisions) was applied with interpretation guidelines (Ding et al., 2016). Results demonstrated: in optional Phase 1 (relevance), all reviews were highly relevant; in Phase 2, Domain 1 (eligibility criteria) showed high concern for bias in five reviews (31%) versus low concern in 11 (69%); Domain 2 (study identification/selection) had high concern in 14 reviews (87%) and unclear concern in 2 (13%); Domain 3 (data collection/study appraisal) revealed high concern in 7 (44%), low concern in 6 (38%), and unclear concern in 3 (19%); Domain 4 (synthesis/findings) exhibited high concern across all 16 reviews (100%); ultimately in Phase 3 (overall bias judgment), all reviews were rated high risk (Figure 6, Supplementary Appendix Tables 4, 5).

#### 3.6.4 GRADE system

**FIGURE 7:**
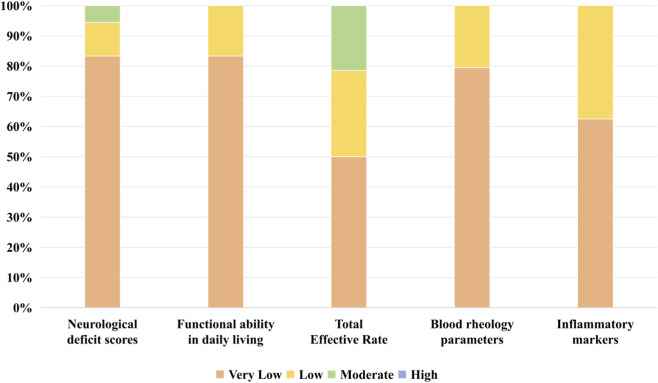
Qualities of the evidence measuring outcomes rated by the GRADE system.

Using the GRADE framework with interpretation guidelines (Wang and He, 2018), researchers assessed the quality of evidence for 102 outcome indicators comprising 125 distinct evidence bodies. Overall, evidence quality distribution was: 7 (5.6%) moderate, 21 (16.8%) low, and 97 (77.6%) very low. For primary outcomes: neurological deficit scores had 18 evidence bodies (1 moderate, 2 low, and 15 very low); activities of daily living showed 6 evidence bodies (1 low and 5 very low); clinical response rate comprised 14 evidence bodies (3 moderate, 4 low, and 7 very low). For secondary outcomes: fibrinogen levels included 8 evidence bodies (3 low and 5 very low). Outcomes with fewer than 5 evidence bodies per indicator are not detailed (Figure 7, Supplementary Appendix Table 6).

## 4 Discussion

### 4.1 Main outcome indicators

#### 4.1.1 Efficacy-related indices

Shuxuetong injection demonstrated statistically confirmed efficacy and safety in primary outcomes. SXT + CT significantly improved neurological deficit and daily living ability compared to CT alone, with higher total response rates. SXT showed superior efficacy in the main outcomes versus other Chinese medicine/biomedicine injections. However, two studies reported comparable effects on CSS score improvement between SXT and LMW, LMWH calcium, or troxerutin. One earlier study found similar clinical efficacy between SXT and other Chinese medicine/biomedicine injections. This non-significance may be attributed to a limited sample size.

A prespecified subgroup analysis stratified by post-treatment assessment timepoints (24 h, 48 h, 7 d, and 14 d) revealed significantly lower NIHSS scores at day 14 compared with earlier intervals, suggesting potential superior efficacy of a 14-day course of SXT + rt-PA. This aligns with expert consensus recommending a 7–14-day treatment duration. However, the validity of these findings is limited by disproportionate study weighting: the 14-day subgroup incorporated data from six RCTs, whereas other timepoints relied on only one to two RCTs each, potentially reducing statistical power at earlier assessments. Additionally, the inability to blind assessors and heterogeneity in baseline characteristics across subgroups (derived from distinct patient populations) may compromise inter-group comparability. Thus, the observed temporal efficacy pattern necessitates verification through prospective trials with standardized outcome assessments and sufficient subgroup sample sizes.

#### 4.1.2 General safety aspects and adverse events

Safety testing of Shuxuetong injection confirms that the formulation lacks mutagenicity, teratogenicity, hemolytic activity, or reproductive toxicity and causes no irritation to blood vessels or muscle tissue at the injection site. The most common AEs reported for Shuxuetong injection are allergic reactions, with skin and skin appendage disorders being the predominant manifestation. Clinical manifestations include skin itching, various allergic rashes, and skin swelling. Respiratory system disorders were the second most frequent, primarily presenting as dyspnea, shortness of breath, and laryngeal edema. Systemic reactions were the third most frequent, clinically presenting as fever, fatigue, and chills. AEs typically occur early, with the majority occurring within 1 h and most frequently within 30 min of administration. The majority of AEs resolved or improved with symptomatic treatment, such as antiallergic therapy, with most patients experiencing resolution within 1 h. No association was observed with the concomitant use of aspirin, ozagrel sodium, prostaglandin E1, edaravone, or methylcobalamin (Mudanjiang Youbo Pharmaceutical Co, 2020).

This overview indicates that most ADRs associated with Shuxuetong injection were mild, consistent with real-world data showing an overall incidence of 4.23‰ (Chen et al., 2022). Notably, one study reported gastrointestinal bleeding and symptomatic intracranial hemorrhage (sICH). These events likely reflect complications of thrombolysis or interactions with anticoagulants, rather than Shuxuetong-specific toxicity. Another study noted infusion-rate-dependent reactions (dizziness and palpitations) that were reversible upon slowing the infusion, corroborating the identified risk factors.

Overall, Shuxuetong injection exhibits a reassuring safety profile: ADRs are predominantly mild, rapidly reversible upon discontinuation, and lack drug-related mortality.

### 4.2 Secondary outcome indicators

For secondary outcomes, comparisons between SXT + CT versus CT alone or other Chinese medicine/biomedicine injections demonstrated statistically significant improvements in most studies. Some non-significant findings in select comparisons likely reflect limited statistical power due to small sample sizes (inability to reject null hypotheses despite clinically meaningful differences) and methodological heterogeneity from inconsistent control interventions across trials.

### 4.3 The methodological quality assessment results

#### 4.3.1 Summary of the most common methodological flaws

According to AMSTAR-2 Item 2, none of the included SRs had a prospectively registered protocol. The primary aims of prospectively registering an SR protocol are to reduce bias, increase transparency, and bolster the review’s credibility. Additionally, registration helps prevent unintended duplication of research efforts, as it allows researchers to identify ongoing or completed reviews in the same field before commencing a new one, thus promoting efficient allocation of academic resources. The absence of a pre-registered protocol increases the risk of selective reporting bias, where only favorable or statistically significant outcomes are published, which can lead to biased estimates of an intervention’s true effect. This practice undermines the methodological rigor and transparency of the research process, ultimately compromising the reliability of the conclusions and hindering independent review and validation.

According to AMSTAR-2 Item 3, most SRs mentioned the type of research included in the Population (P), Intervention (I), Comparison (C), and Outcome (O) (PICO) section. However, none explicitly stated the rationale for selecting that specific study type. This may imply selective reporting or selective inclusion of studies with a high risk or favorable outcomes, potentially distorting conclusions. Such omissions may suggest biased selection of SRs, which could lead to overestimation of intervention effects. However, all 16 SRs included in this study selected RCTs (including quasi-RCTs) as the study type. Given that RCTs represent the highest level of evidence in evidence-based medicine, we believe that, despite the lack of full reporting of the rationale for selection, this choice may reflect researchers' high standards for the quality of evidence in the original studies to a certain extent. Nevertheless, according to the AMSTAR-2 tool, which is a measurement tool for assessing the methodological quality of systematic reviews, the reporting deficiency in question constitutes a methodological flaw that affects methodological transparency and the level of evidence. Consequently, all studies received a “No” rating for item three of the AMSTAR-2 checklist, which evaluates specific aspects of systematic review reporting.

According to AMSTAR-2 Item 4, a “partial yes” rating was assigned to 75% of the included systematic reviews because they did not explicitly report whether grey literature was searched. Grey literature, conventionally defined as reports (pre-prints, preliminary progress, and advanced reports, technical reports, statistical reports, memoranda, state-of-the art reports, market research reports, etc.), theses, conference proceedings, technical specifications and standards, non-commercial translations, bibliographies, technical and commercial documentation, and official documents not published commercially (primarily government reports and documents), can contain valuable data not available in traditional journals (Alberani et al., 1990). Failure to search for and incorporate such sources may lead to the omission of critical evidence, thereby introducing both selection bias and database (indexing) bias into the SR. Additionally, grey literature is often published more rapidly than peer-reviewed journal articles because it bypasses lengthy commercial publication processes. Therefore, excluding it may cause the review to miss the most current data, cutting-edge research, or timely reports on emerging trends.

According to AMSTAR-2 Item 7, the included studies did not provide detailed lists of excluded studies; most reported only the number and reasons for exclusion through PRISMA flowcharts. SRs should comprehensively report the list of potentially relevant studies that were excluded, along with the reasons for their exclusion, to ensure transparency in the literature screening process. Excluding studies based on risk of bias may introduce selection bias, and the absence of detailed exclusion lists may limit the reproducibility and verifiability of the review’s findings.

According to AMSTAR-2 Item 9, 50% of the included studies were rated “partial yes” due to incomplete reporting of specific aspects (e.g., whether the allocation sequence was truly random or the presence of selective reporting). This may be attributed to the lack of standardized tools for assessing risk of bias, leading to gaps in reporting. The use of non-standardized tools may introduce subjectivity in bias risk assessment across SRs, potentially compromising the reliability of evaluation outcomes.

According to AMSTAR-2 Item 10, a high proportion (93.8%) of the included studies failed to report their funding sources. Transparent disclosure of whether a study received funding and the source of that funding is essential, as it enables the assessment of potential conflicts of interest that might have biased the study’s design, execution, or reporting. Consequently, such disclosure is critical for judging whether the reported efficacy outcomes could have been overstated. Studies that fail to disclose funding sources may conceal conflicts of interest, potentially compromising the integrity of research by introducing bias through mechanisms such as selective reporting, data manipulation, or biased analytical approaches. Such bias can undermine the credibility of findings and the reliability of evidence, particularly in contexts in which transparency and methodological rigor are paramount.

According to AMSTAR-2 Item 12, when pooled effect estimates are based on RCTs with varying levels of risk of bias, 87.5% of the included studies failed to evaluate the potential impact of bias on the overall effect estimate. The distribution of effect sizes may differ between studies with high and low risk of bias. When RCTs with varying bias levels are pooled without distinction, the overestimated effect values from high-risk studies may inflate the overall pooled effect, introducing bias in the conclusions (Table 3).

**TABLE 3:** Summary of methodological limitations according to the AMSTAR 2 tool.

Item#	Item detail	Yes	Partial yes	No	Methodological flaws	Potential impact
2	Did the report of the review contain an explicit statement that the review methods were established prior to the conduct of the review, and did the report justify any significant deviations from the protocol?	0%	0%	**100%**	Lack of prospectively registered protocol	Distortion of true effect estimates Compromised reproducibility and integrity Inefficient allocation and duplication of academic resources
3	Did the review authors explain their selection of the study designs for inclusion in the review?	0%	0%	**100%**	Lack of rationale for selecting the type of research	Intervention efficacy (effect inflation)
4	Did the review authors use a comprehensive literature search strategy?	25%	**75%**	0%	Lack of retrieval of grey literature	Risk of publication bias Risk of database or indexing bias Reduced comprehensiveness and representativeness of the evidence base Compromised timeliness and relevance, especially for emerging studies
7	Did the review authors provide a list of excluded studies and justify the exclusions?	0%	0%	**100%**	Lack of detailed lists of excluded studies and the reasons for their exclusion	Risk of selection bias Limitation on reproducibility and verifiability
9	Did the review authors use a satisfactory technique for assessing the risk of bias (RoB) in individual studies that were included in the review?	43.80%	**50%**	6.20%	Incomplete reporting of specific aspects (e.g., whether the allocation sequence was truly random or the presence of selective reporting)	Inconsistency, reduced comparability Hindered reproducibility of results Introduces subjectivity and arbitrariness into the assessment process
10	Did the review authors report on the sources of funding for the studies included in the review?	6.20%	0%	**93.80%**	Lack of reporting the funding for the RCTs included in SRs	Risk of selection bias Compromised data integrity
12	If meta-analysis was performed, did the review authors assess the potential impact of RoB in individual studies on the results of the meta-analysis or other evidence synthesis?	12.50%	0%	**87.50%**	Failed to evaluate the potential impact of bias on the overall effect estimate when pooled effect estimates are based on RCTs with varying levels of risk of bias	Demonstrate inflated (overestimated) effect sizes Biased estimates of treatment effects

In this study, we defined that if more than 50% of the included SRs were rated as “partially yes” or “no” for a given item, the included SRs were considered to have an overall high risk of bias for that item, which merits specific discussion in the main text and tables. These data are shown in bold in Table 3.

According to the PRISMA 2020 Abstract Checklist, all included studies had incomplete abstracts, with funding and registration details being particularly underreported. Assessment against PRISMA 2020 Checklist Item 25 and AMSTAR-2 Item 16 revealed that more than 62.5% of the included studies reported funding sources and/or declarations of no conflicts of interest within their main text. Nevertheless, because this information was omitted from the abstracts, these studies were still assigned a rating of “partial yes” for Item 2. The issue of missing prior protocol registration was explicitly addressed under the assessment of AMSTAR-2 Item 2.

According to PRISMA 2020 Checklist Item 5, the included studies did not report their methods for grouping studies during the synthesis phase. Subgroup analysis is a crucial methodological approach to examine whether intervention effects differ across various patient characteristics, intervention protocols, or study designs. The omission of reporting prespecified subgroup analyses can lead to selective reporting bias. In such bias, researchers might selectively present only those subgroups that demonstrate statistically significant or expected outcomes, consequently distorting the overall conclusions of the research.

According to PRISMA 2020 Checklist Item 20c, half of the included studies investigated potential sources of heterogeneity. Heterogeneity often arises from methodological biases in study design, conduct, or reporting. Failing to explore these sources can lead SRs to mistakenly conclude that an exaggerated or underestimated effect resulting from flaws like inadequate blinding or allocation concealment is attributable to the intervention itself. Alternatively, heterogeneity can reflect genuine clinical heterogeneity, indicating real variations in treatment effects across different patient populations, dosages, or disease stages. An SR that fails to distinguish between these possibilities may yield overly generalized conclusions of limited practical utility for clinical decision-making (Table 4).

**TABLE 4:** Summary of methodological limitations according to the PRISMA 2020 checklist.

Item#	Checklist item details	Yes	Partial yes	No	Methodological flaws	Potential impact
2	See the PRISMA 2020 for Abstract Checklist (title, objectives, eligibility criteria, information sources, risk of bias, synthesis of results, included studies, synthesis of results, limitations of evidence, interpretation, funding, and registration)	0%	**100%**	0%	Incomplete abstracts with funding and registration details being particularly underreported	Undermines the integrity, transparency, and accessibility of abstracts (the issue of missing prior protocol registration was explicitly addressed under the assessment of AMSTAR-2 Item 2)
5	Specify the inclusion and exclusion criteria for the review and how studies were grouped for the syntheses	0%	**100%**	0%	Failed to report the methods for grouping RCTs during the synthesis phase	Risk of selection bias Misattribution of effects to the intervention due to methodological flaws
6	Specify all databases, registers, websites, organizations, reference lists, and other sources searched or consulted to identify studies. Specify the date when each source was last searched or consulted	0%	**100%**	0%	Lack of retrieval of registration platform, website, organization, and grey literature	Similar problems were explicitly addressed under the assessment of AMSTAR-2 Item 4
7	Present the full search strategies for all databases, registers, and websites, including any filters and limits used	0%	**100%**	0%	Lack of reporting the comprehensive search strategies	Risk of selection bias Compromised reproducibility
10a	List and define all outcomes for which data were sought. Specify whether all results that were compatible with each outcome domain in each study were sought (e.g., for all measures, timepoints, and analyses), and if not, the methods used to decide which results to collect	0%	**100%**	0%	Lack of detailed descriptions of measurement methods, timepoints, or analytical methods	Methodological heterogeneity Inappropriate grouping of disparate data points under a single outcome indicator
10b	List and define all other variables for which data were sought (e.g., participant and intervention characteristics and funding sources). Describe any assumptions made about any missing or unclear information	0%	**100%**	0%	Incomplete reporting of other variables Absence of assumptions for missing or ambiguous information	Introduces reporting bias
11	Specify the methods used to assess risk of bias in the included studies, including details of the tool(s) used, how many reviewers assessed each study, and whether they worked independently, and if applicable, details of automation tools used in the process	12.50%	**81.20%**	6.30%	Absence of duplicate review by two evaluators	Increased risk of bias and error in study selection and data extraction Cumulative amplification of bias across the review pipeline
15	Describe any methods used to assess certainty (or confidence) in the body of evidence for an outcome	25%	0%	**75%**	No reported evidence quality assessment methods	Misleading overestimation of evidence quality Introduces publication bias
16b	Cite studies that might appear to meet the inclusion criteria but were excluded; explain why they were excluded	0%	0%	**100%**	Absence of the excluded eligible study list with rationale	Similar problems were explicitly addressed under the assessment of AMSTAR-2 Item 7
20a	For each synthesis, briefly summarize the characteristics and risk of bias among contributing studies	0%	**100%**	0%	Outcomes, risk of bias, and forest plots not integrated	An incomplete presentation of results hinders a comprehensive assessment
20c	Present the results of all investigations of possible causes of heterogeneity among study results	50%	0%	**50%**	Lack of heterogeneity source investigation	Erroneous attribution of effect Failure to distinguish true clinical variation from methodological artifact Overly generalized and less useful conclusions
21	Present assessments of risk of bias due to missing results (arising from reporting biases) for each synthesis assessed	18.80%	25%	**56.20%**	Publication bias from missing outcomes not assessed Publication bias could not be evaluated as <10 studies were included	Can substantively alter pooled effect estimates, potentially changing the interpretation of findings (Kahale et al., 2020)
22	Present assessments of certainty (or confidence) in the body of evidence for each outcome assessed	25%	0%	**75%**	The quality of evidence for each outcome was not reported	Similar problems were explicitly addressed under the assessment of PRISMA 2020 Item 15
24a	Provide registration information for the review, including the register name and registration number, or state that the review was not registered	0%	0%	**100%**	Lack of prospectively registered protocol	Similar problems were explicitly addressed under the assessment of AMSTAR-2 Item 2
24b	Indicate where the review protocol can be accessed, or state that a protocol was not prepared	0%	0%	**100%**		
24c	Describe and explain any amendments to information provided at registration or in the protocol	0%	0%	**100%**		
26	Declare any competing interests of review authors	12.50%	0%	**87.50%**	Failure to declare SRs’ competing interests	Similar problems were explicitly addressed under the assessment of AMSTAR-2 Item 10
27	Report which of the following are publicly available and where they can be found: template data collection forms; data extracted from included studies; data used for all analyses; analytic code; any other materials used in the review	0%	0%	**100%**	No reporting on data availability and access	Impairs the verification, reproducibility, and critical appraisal of existing research Hinders or halts subsequent research projects and scientific collaboration

In this study, we defined that if more than 50% of the included SRs were rated as “partially yes” or “no” for a given item, the included SRs were considered to have an overall high risk of bias for that item, which merits specific discussion in the main text and tables. These data are shown in bold in Table 4.

#### 4.3.2 Implications for future research

Based on the discussion of the aforementioned methodological limitations, we believe that SR developers urgently need to improve several critical aspects of their work. Key areas for improvement include the implementation of prospective registration, the justification of exclusion criteria, the use of appropriate tools to assess risk of bias and evaluate the quality of outcome evidence, and the exploration and discussion of sources of heterogeneity. Enhancing these areas will make the results of SRs more rigorous and comprehensive and increase the value and clinical applicability of their findings.

### 4.4 Study limitations and prospects

During the analysis of results and quality assessment, several areas for improvement were identified.

Following the outcome indicators adopted in the included reviews, the total response rate was designated as the main outcome in this study. However, its designation is controversial due to its limited utility in guiding clinical decisions. The total response rate, derived from the proportion of cases categorized as “cured,” “markedly improved,” or “improved,” entails loss of information when converting continuous outcomes to ordinal scales. This reduces statistical power in hypothesis testing. Furthermore, heterogeneity arises from inconsistent terminology, non-uniform efficacy scales, and variable measurement timepoints across studies. Such heterogeneity may compromise the validity of pooled effect estimates in meta-analyses (Li et al., 2020).

Outcome measures reflecting disease trajectory are notably scarce in the included studies, limiting the translation of these findings to clinical applicability. To address this gap, we recommend incorporating quantitative symptom scales (e.g., Modified Rankin Scale [mRS] and Glasgow Coma Scale [GCS]), disease progression endpoints (e.g., mortality and recurrence rates), direct imaging assessments of lesion evolution (e.g., infarct volume), etc. Notably, only one included study reported mortality data, which showed no significant difference. It underscores some room for improvement in studying long-term functional outcomes of IS within existing RCTs and highlights the need for future trials to adopt standardized core outcome sets (COS) for comprehensive clinical relevance.

According to evidence-based methodology principles, insufficient methodological detail does not automatically warrant the exclusion of an original study. However, such a lack requires stringent caution in data synthesis and in interpreting results. Some SRs were limited by early publication dates and insufficient information in the original reports, preventing us from assessing whether researchers had adequately controlled for bias. Consequently, we were unable to assess the risk of selection bias. The relationship between these SRs’ positive results and their methodological limitations remains unclear. Therefore, we exercised greater caution in evaluating the evidence for efficacy in the discussion section. With the advancement of evidence-based medicine, SRs have become increasingly comprehensive and rigorous in their design, demonstrating gradual improvements in methodological quality and thus offering valuable reference points. Numerous SRs have addressed this question to date. Moving beyond a sole focus on treatment efficacy, this study aims to synthesize all existing evidence while critically appraising the methodological quality of these prior reviews using established assessment tools. By identifying methodological limitations in the available SRs, we seek to provide clearer methodological guidance for the design of future studies. It is intended to help generate more reliable, high-level evidence to inform practice in related fields.

Therefore, we believe these SRs can still be used to evaluate the efficacy of Shuxuetong injection for IS, although they are subject to significant limitations. They offer only preliminary findings that should be interpreted with caution, rather than definitive evidence of efficacy. The value of this study lies in systematically reviewing existing data, highlighting widespread methodological limitations, and guiding future research toward areas for improvement.

## 5 Conclusion

Based on the analysis, this overview of systematic reviews and meta-analyses suggests that Shuxuetong injection is effective and safe for the treatment of IS. However, the overall low methodological quality of included studies undermines the certainty of these findings. Further high-quality RCTs are warranted to strengthen the evidence base, and the rigor of study design and completeness of reporting require improved standardization across studies.

## Data Availability

The original contributions presented in the study are included in the article/Supplementary Material; further inquiries can be directed to the corresponding authors.
