# Supplementary material for: Shuxuetong injection for ischemic stroke: an overview of systematic reviews using different methodological quality assessment tools
Source: Front Pharmacol. 2026 Apr 13;17:1723582. doi: 10.3389/fphar.2026.1723582 (PMC13111222; doi:10.3389/fphar.2026.1723582)
Supplement: Supplementary file 1 [file Table1.docx]

Table 1 Overlap matrix of included primary studies

| Literature Overlap Matrix | Zhang et al.  (2024) | Li et al.  (2023) | Zhao et al.  (2022) | Xiang et al.  (2017) | Li et al.  (2017) | Chen et al.  (2016) | Ma (2015) | Wang (2013) | Zhang et al.  (2012) | Ding et al.  (2011) | Lei (2010) | Ma and Li  (2010) | Wu et al.  (2010) | Su et al.  (2010) | Li and Miao  (2007) | Liu et al.  (2006) |
| --- | --- | --- | --- | --- | --- | --- | --- | --- | --- | --- | --- | --- | --- | --- | --- | --- |
| Ha et al.  (2020) | x |  |  |  |  |  |  |  |  |  |  |  |  |  |  |  |
| Zhao et al.  (2019) | x |  |  |  |  |  |  |  |  |  |  |  |  |  |  |  |
| Fang et al.  (2019) | x |  |  |  |  |  |  |  |  |  |  |  |  |  |  |  |
| Jin et al.  (2018) | x |  |  |  |  |  |  |  |  |  |  |  |  |  |  |  |
| Yu, R. et al.  (2017) | x |  |  |  |  |  |  |  |  |  |  |  |  |  |  |  |
| Zhang et al.  (2018) | x |  |  |  |  |  |  |  |  |  |  |  |  |  |  |  |
| Yu et al.  (2021) | x |  |  |  |  |  |  |  |  |  |  |  |  |  |  |  |
| Guo et al.  (2022) | x |  |  |  |  |  |  |  |  |  |  |  |  |  |  |  |
| Zhang, X. et al. (2005a) |  | x |  |  |  |  |  |  |  |  |  | x |  |  |  |  |
| Xie et al.  (2006) |  | x |  |  |  |  |  |  |  |  |  |  |  |  |  |  |
| Dai et al.  (2006) |  | x |  |  |  |  |  |  |  |  |  |  |  |  |  |  |
| Li et al.  (2006) |  | x |  |  |  |  |  |  |  |  |  | x |  |  |  |  |
| Wang et al.  (2006) |  | x |  |  |  |  |  |  |  |  |  | x | x |  |  |  |
| He et al.  (2008) |  | x |  |  |  |  |  |  |  |  |  | x |  |  |  |  |
| Yang et al.  (2009) |  | x |  |  |  |  |  |  |  |  |  |  | x |  |  |  |
| Liu et al.  (2010) |  | x |  |  |  |  |  |  |  |  |  |  |  |  |  |  |
| Zhou et al.  (2010) |  | x |  |  |  |  |  |  |  |  |  |  |  |  |  |  |
| Chen et al.  (2011) |  | x |  |  |  |  |  |  |  |  |  |  |  |  |  |  |
| Huang et al.  (2011) |  | x |  |  |  |  |  |  |  |  |  |  |  |  |  |  |
| Li et al.  (2011) |  | x |  |  |  |  |  |  |  |  |  |  |  |  |  |  |
| Xie et al.  (2011) |  | x |  |  |  |  |  |  |  |  |  |  |  |  |  |  |
| Zhen et al.  (2011) |  | x |  |  |  |  |  |  |  |  |  |  |  |  |  |  |
| Yan et al.  (2011) |  | x |  |  |  |  |  |  |  |  |  |  |  |  |  |  |
| Zou et al.  (2013) |  | x |  |  |  |  |  |  |  |  |  |  |  |  |  |  |
| Lv et al.  (2013) |  | x |  |  |  |  |  |  |  |  |  |  |  |  |  |  |
| Guo, L. et al.  (2014) |  | x |  |  |  |  |  |  |  |  |  |  |  |  |  |  |
| Liu et al.  (2014) |  | x | x |  |  |  |  |  |  |  |  |  |  |  |  |  |
| Zang et al.  (2014) |  | x |  |  |  |  |  |  |  |  |  |  |  |  |  |  |
| Wu et al.  (2014) |  | x |  |  |  |  |  |  |  |  |  |  |  |  |  |  |
| Zhao et al.  (2015) |  | x |  |  |  |  |  |  |  |  |  |  |  |  |  |  |
| Du et al.  (2015) |  | x |  |  |  |  |  |  |  |  |  |  |  |  |  |  |
| Sun, L. et al.  (2015) |  | x |  |  |  |  |  |  |  |  |  |  |  |  |  |  |
| Sun, K. et al.  (2015) |  | x |  |  |  |  |  |  |  |  |  |  |  |  |  |  |
| Xin et al.  (2016) |  | x |  |  |  |  |  |  |  |  |  |  |  |  |  |  |
| Wang et al.  (2016) |  | x |  |  |  |  |  |  |  |  |  |  |  |  |  |  |
| Cao et al.  (2017) |  | x |  |  |  |  |  |  |  |  |  |  |  |  |  |  |
| Yu, D. et al.  (2017) |  | x |  |  |  |  |  |  |  |  |  |  |  |  |  |  |
| Tan et al.  (2017) |  | x |  |  |  |  |  |  |  |  |  |  |  |  |  |  |
| Jiao et al.  (2018) |  | x |  |  |  |  |  |  |  |  |  |  |  |  |  |  |
| Tan et al.  (2021) |  |  | x |  |  |  |  |  |  |  |  |  |  |  |  |  |
| Wang et al.  (2018) |  |  | x |  |  |  |  |  |  |  |  |  |  |  |  |  |
| Lv et al.  (2017) |  |  | x |  |  |  |  |  |  |  |  |  |  |  |  |  |
| He et al.  (2017) |  |  | x |  |  |  |  |  |  |  |  |  |  |  |  |  |
| Wang et al.  (2017) |  |  | x |  |  |  |  |  |  |  |  |  |  |  |  |  |
| Zeng et al.  (2016) |  |  | x |  |  |  |  |  |  |  |  |  |  |  |  |  |
| Li et al.  (2016) |  |  | x |  |  |  |  |  |  |  |  |  |  |  |  |  |
| Wu et al.  (2015) |  |  | x |  |  |  |  |  |  |  |  |  |  |  |  |  |
| Zhang, S. et al. (2014) |  |  | x |  |  |  |  |  |  |  |  |  |  |  |  |  |
| Guo, Y. et al.  (2014) |  |  | x |  |  |  |  |  |  |  |  |  |  |  |  |  |
| Yu et al.  (2013) |  |  | x |  |  |  |  |  |  |  |  |  |  |  |  |  |
| Xi et al.  (2012) |  |  | x |  |  |  |  |  | x |  |  |  |  |  |  |  |
| Li et al.  (2012) |  |  | x |  |  |  |  |  | x |  |  |  |  |  |  |  |
| Lian et al.  (2012) |  |  | x |  |  |  |  |  | x |  |  |  |  |  |  |  |
| Xia et al.  (2011) |  |  | x |  |  |  |  |  | x |  |  |  |  |  |  |  |
| Zhao et al.  (2011) |  |  | x |  |  |  |  |  | x |  |  |  |  |  |  |  |
| Huang et al.  (2011) |  |  | x |  |  |  |  |  | x |  |  |  |  |  |  |  |
| Liu et al.  (2010) |  |  | x |  |  |  |  |  |  |  |  |  |  |  |  |  |
| Jin et al.  (2009) |  |  | x |  |  |  |  |  |  |  |  |  |  |  |  |  |
| Yang et al.  (2008) |  |  | x |  |  |  |  |  | x |  |  |  |  |  |  |  |
| Zhang et al.  (2008) |  |  | x |  |  |  |  |  | x |  |  | x |  |  |  |  |
| Wang et al.  (2009) |  |  |  | x |  |  |  |  |  |  |  |  |  |  |  |  |
| Liu et al.  (2012) |  |  |  | x |  |  |  |  |  |  |  |  |  |  |  |  |
| Shen et al.  (2012) |  |  |  | x |  |  |  |  |  |  |  |  |  |  |  |  |
| Jiang et al.  (2013) |  |  |  | x |  |  |  |  |  |  |  |  |  |  |  |  |
| Li et al.  (2015) |  |  |  | x |  |  |  |  |  |  |  |  |  |  |  |  |
| Liang et al.  (2015) |  |  |  | x |  |  |  |  |  |  |  |  |  |  |  |  |
| Tan et al.  (2015) |  |  |  | x |  |  |  |  |  |  |  |  |  |  |  |  |
| Liu et al.  (2016) |  |  |  | x |  |  |  |  |  |  |  |  |  |  |  |  |
| Tang et al.  (2007) |  |  |  |  | x |  |  |  |  |  |  |  |  |  |  |  |
| Zhang, P. et al.  (2014) |  |  |  |  | x |  |  |  |  |  |  |  |  |  |  |  |
| Zeng et al.  (2016) |  |  |  |  | x |  |  |  |  |  |  |  |  |  |  |  |
| Wang et al.  (2012) |  |  |  |  | x |  |  |  |  |  |  |  |  |  |  |  |
| Sun, B. et al.  (2015) |  |  |  |  | x |  |  |  |  |  |  |  |  |  |  |  |
| Zeng et al.  (2015) |  |  |  |  | x |  |  |  |  |  |  |  |  |  |  |  |
| Li, B. et al.  (2010) |  |  |  |  | x |  |  |  |  |  |  |  |  |  |  |  |
| Wen et al.  (2012) |  |  |  |  | x |  |  |  |  |  |  |  |  |  |  |  |
| Xiong et al.  (2013) |  |  |  |  | x |  |  |  |  |  |  |  |  |  |  |  |
| Han et al.  (2012) |  |  |  |  | x |  |  |  |  |  |  |  |  |  |  |  |
| Wei et al.  (2009) |  |  |  |  | x |  |  |  |  |  |  |  |  |  |  |  |
| Ling et al.  (2007) |  |  |  |  |  | x |  |  |  |  |  |  |  |  |  |  |
| Li et al.  (2009) |  |  |  |  |  | x |  |  |  |  |  |  |  | x |  |  |
| Wu et al.  (2010) |  |  |  |  |  | x | x | x |  |  |  |  |  |  |  |  |
| Zhou et al.  (2004) |  |  |  |  |  | x |  |  |  |  |  |  |  |  |  |  |
| Zhang et al.  (2011) |  |  |  |  |  | x |  |  |  |  |  |  |  |  |  |  |
| Xu et al.  (2007) |  |  |  |  |  | x |  |  |  |  |  |  |  |  |  |  |
| Fang et al.  (2015) |  |  |  |  |  | x |  |  |  |  |  |  |  |  |  |  |
| Fang et al.  (2011) |  |  |  |  |  | x |  |  |  |  |  |  |  |  |  |  |
| Li, Y. et al.  (2010) |  |  |  |  |  | x |  |  |  |  |  |  |  |  |  |  |
| Cai et al.  (2006) |  |  |  |  |  | x | x |  |  |  |  |  |  |  |  |  |
| Lai et al.  (2008) |  |  |  |  |  | x |  |  |  |  |  |  | x |  |  |  |
| Chen et al.  (2010) |  |  |  |  |  | x |  |  |  |  |  |  |  |  |  |  |
| Chen et al.  (2015) |  |  |  |  |  | x |  |  |  |  |  |  |  |  |  |  |
| Lv et al.  (2009) |  |  |  |  |  |  | x |  |  |  | x |  |  |  |  |  |
| Wu et al.  (2011) |  |  |  |  |  |  | x |  |  |  |  |  |  |  |  |  |
| Zhou et al.  (2007) |  |  |  |  |  |  | x | x |  |  |  |  |  |  |  |  |
| Zhang, H. et al. (2005) |  |  |  |  |  |  | x | x |  | x |  |  |  |  |  | x |
| Zeng et al.  (2005) |  |  |  |  |  |  | x |  |  |  |  |  |  |  |  |  |
| Li, Y. et al.  (2008) |  |  |  |  |  |  | x |  |  |  |  |  |  |  |  |  |
| Yang et al.  (2006) |  |  |  |  |  |  | x |  |  |  |  |  | x |  |  |  |
| Xiong et al.  (2011) |  |  |  |  |  |  | x | x |  |  |  |  |  |  |  |  |
| Niu et al.  (2005) |  |  |  |  |  |  | x |  |  |  |  |  |  |  |  |  |
| Wang et al.  (2005) |  |  |  |  |  |  | x |  |  |  |  |  |  |  |  |  |
| Xu et al.  (2006) |  |  |  |  |  |  | x |  |  |  |  |  |  |  |  |  |
| Zhao et al.  (2006) |  |  |  |  |  |  | x |  |  |  |  |  |  |  |  |  |
| Xin et al.  (2010) |  |  |  |  |  |  | x |  |  |  |  |  |  |  |  |  |
| Li, N. et al.  (2007) |  |  |  |  |  |  |  | x |  | x |  |  | x |  |  |  |
| Fang et al.  (2008) |  |  |  |  |  |  |  | x |  | x | x |  | x |  |  |  |
| Bai et al.  (2010) |  |  |  |  |  |  |  | x |  |  |  |  |  |  |  |  |
| Cheng et al.  (2010) |  |  |  |  |  |  |  | x |  |  |  |  |  |  |  |  |
| Guo et al.  (2010) |  |  |  |  |  |  |  | x |  |  |  |  |  |  |  |  |
| Sun et al.  (2002) |  |  |  |  |  |  |  | x |  |  |  |  |  |  |  | x |
| Wang et al.  (2004) |  |  |  |  |  |  |  | x |  | x |  |  |  |  |  | x |
| Wang et al.  (2006) |  |  |  |  |  |  |  | x |  | x |  |  | x |  |  |  |
| Huang et al.  (2010) |  |  |  |  |  |  |  | x |  |  |  |  |  |  |  |  |
| Ji et al.  (2006) |  |  |  |  |  |  |  | x |  |  |  |  |  |  |  |  |
| Yan et al.  (2008) |  |  |  |  |  |  |  | x |  | x |  |  | x |  |  |  |
| Ye et al.  (2005) |  |  |  |  |  |  |  | x |  | x |  |  |  |  |  |  |
| Qian et al.  (2006) |  |  |  |  |  |  |  | x |  | x |  |  |  |  |  |  |
| Chen et al.  (2004) |  |  |  |  |  |  |  | x |  |  |  |  |  |  |  | x |
| Zhang et al.  (2004) |  |  |  |  |  |  |  |  | x |  |  |  |  |  |  |  |
| Dong et al.  (2005) |  |  |  |  |  |  |  |  | x |  |  |  |  |  |  |  |
| Guo et al.  (2012) |  |  |  |  |  |  |  |  | x |  |  |  |  |  |  |  |
| Chen et al.  (2007) |  |  |  |  |  |  |  |  |  | x |  |  | x |  |  |  |
| Li, J. et al.  (2007) |  |  |  |  |  |  |  |  |  | x |  |  |  |  |  |  |
| Lei et al.  (2006) |  |  |  |  |  |  |  |  |  |  | x |  |  |  |  |  |
| Jiang et al.  (2006) |  |  |  |  |  |  |  |  |  |  | x |  |  |  |  |  |
| Chen et al.  (2009) |  |  |  |  |  |  |  |  |  |  | x |  |  |  |  |  |
| Zhou et al.  (2009) |  |  |  |  |  |  |  |  |  |  | x |  | x |  |  |  |
| Wang et al.  (2005) |  |  |  |  |  |  |  |  |  |  | x |  |  |  |  |  |
| Song et al.  (2006) |  |  |  |  |  |  |  |  |  |  | x |  |  |  |  |  |
| Sun et al.  (2005) |  |  |  |  |  |  |  |  |  |  | x |  | x |  |  | x |
| Yang et al.  (2004) |  |  |  |  |  |  |  |  |  |  | x |  | x |  |  | x |
| Zhang et al.  (2005b) |  |  |  |  |  |  |  |  |  |  | x |  |  |  | x |  |
| Shi et al.  (2008) |  |  |  |  |  |  |  |  |  |  | x |  | x |  |  |  |
| Zeng et al.  (2008) |  |  |  |  |  |  |  |  |  |  | x |  | x |  |  |  |
| Bao et al.  (2008) |  |  |  |  |  |  |  |  |  |  |  | x |  |  |  |  |
| Cao et al.  (2008) |  |  |  |  |  |  |  |  |  |  |  | x |  |  |  |  |
| Chen et al.  (2008) |  |  |  |  |  |  |  |  |  |  |  | x |  |  |  |  |
| Gu et al.  (2007) |  |  |  |  |  |  |  |  |  |  |  | x |  |  |  |  |
| Lin et al.  (2007) |  |  |  |  |  |  |  |  |  |  |  | x |  |  |  |  |
| Pan et al.  (2007) |  |  |  |  |  |  |  |  |  |  |  | x |  |  |  |  |
| Pang et al.  (2006) |  |  |  |  |  |  |  |  |  |  |  | x |  |  |  |  |
| Quan et al.  (2005) |  |  |  |  |  |  |  |  |  |  |  | x |  |  |  |  |
| Sun et al.  (2008) |  |  |  |  |  |  |  |  |  |  |  | x |  |  |  |  |
| Wang et al.  (2004) |  |  |  |  |  |  |  |  |  |  |  | x |  |  | x |  |
| Xie et al.  (2006) |  |  |  |  |  |  |  |  |  |  |  | x |  |  |  |  |
| Zhang, J. et al.  (2008) |  |  |  |  |  |  |  |  |  |  |  | x |  |  |  |  |
| Zhang, W. et al. (2008) |  |  |  |  |  |  |  |  |  |  |  | x |  | x |  |  |
| Zhao et al.  (2008) |  |  |  |  |  |  |  |  |  |  |  | x | x |  |  |  |
| Zhou et al.  (2007) |  |  |  |  |  |  |  |  |  |  |  | x | x |  |  |  |
| Ye et al.  (2006) |  |  |  |  |  |  |  |  |  |  |  | x |  |  |  |  |
| Wang et al.  (2004) |  |  |  |  |  |  |  |  |  |  |  | x |  |  | x | x |
| Wang et al.  (2008) |  |  |  |  |  |  |  |  |  |  |  | x |  |  |  |  |
| Zhang, J. et al.  (2005) |  |  |  |  |  |  |  |  |  |  |  | x |  |  |  |  |
| Geng et al.  (2004) |  |  |  |  |  |  |  |  |  |  |  |  | x |  |  |  |
| Huang et al.  (2004) |  |  |  |  |  |  |  |  |  |  |  |  | x |  |  |  |
| Yin et al.  (2004) |  |  |  |  |  |  |  |  |  |  |  |  | x |  |  |  |
| Feng et al.  (2005) |  |  |  |  |  |  |  |  |  |  |  |  | x |  |  |  |
| Song et al.  (2005) |  |  |  |  |  |  |  |  |  |  |  |  | x |  |  |  |
| Dai et al.  (2006) |  |  |  |  |  |  |  |  |  |  |  |  | x |  |  |  |
| He et al.  (2007) |  |  |  |  |  |  |  |  |  |  |  |  | x |  |  |  |
| Xiao et al.  (2007) |  |  |  |  |  |  |  |  |  |  |  |  | x |  |  |  |
| Li, Z. et al.  (2008) |  |  |  |  |  |  |  |  |  |  |  |  | x |  |  |  |
| Tu et al.  (2008) |  |  |  |  |  |  |  |  |  |  |  |  | x |  |  |  |
| Fang et al.  (2009) |  |  |  |  |  |  |  |  |  |  |  |  | x |  |  |  |
| Jian et al.  (2009) |  |  |  |  |  |  |  |  |  |  |  |  | x |  |  |  |
| Gan et al.  (2009) |  |  |  |  |  |  |  |  |  |  |  |  |  | x |  |  |
| Zhou et al.  (2008) |  |  |  |  |  |  |  |  |  |  |  |  |  | x |  |  |
| Wang et al.  (2006) |  |  |  |  |  |  |  |  |  |  |  |  |  | x |  |  |
| Huang et al.  (2008) |  |  |  |  |  |  |  |  |  |  |  |  |  | x |  |  |
| Zhang, N. et al. (2008) |  |  |  |  |  |  |  |  |  |  |  |  |  | x |  |  |
| Qiu et al.  (2009) |  |  |  |  |  |  |  |  |  |  |  |  |  | x |  |  |
| Liu et al.  (2009) |  |  |  |  |  |  |  |  |  |  |  |  |  | x |  |  |
| Zhao et al.  (2009) |  |  |  |  |  |  |  |  |  |  |  |  |  | x |  |  |
| Huang et al.  (2009) |  |  |  |  |  |  |  |  |  |  |  |  |  | x |  |  |
| Zhang, X. et al.  (2005c) | |  |  |  |  |  |  |  |  |  |  |  |  |  | x |  |
| Liu et al.  (2005) |  |  |  |  |  |  |  |  |  |  |  |  |  |  |  | x |
| Yin et al.  (2005) |  |  |  |  |  |  |  |  |  |  |  |  |  |  |  | x |
| Luo et al.  (2004) |  |  |  |  |  |  |  |  |  |  |  |  |  |  |  | x |
| Sun et al.  (2003) |  |  |  |  |  |  |  |  |  |  |  |  |  |  |  | x |

Table 2 Methodological quality of included SRs assessed using the AMSTAR 2 tool

| Review  (Year) | 1 | 2* | 3 | 4* | 5 | 6 | 7* | 8 | 9* | 10 | 11* | 12 | 13* | 14 | 15* | 16 | Y | PY | N | Quality level |
| --- | --- | --- | --- | --- | --- | --- | --- | --- | --- | --- | --- | --- | --- | --- | --- | --- | --- | --- | --- | --- |
| Zhang et al.  (2024) | Y | N | N | PY | Y | Y | N | PY | Y | N | Y | N | Y | Y | Y | Y | 9 | 2 | 5 | Critically low |
| Li et al.  (2023) | Y | N | N | PY | Y | Y | N | PY | Y | Y | Y | N | N | Y | Y | Y | 9 | 2 | 5 | Critically low |
| Zhao et al.  (2022) | Y | N | N | PY | Y | N | N | Y | Y | N | Y | N | Y | Y | Y | Y | 9 | 1 | 6 | Critically low |
| Xiang et al.  (2017) | Y | N | N | PY | Y | N | N | Y | Y | N | Y | N | Y | Y | Y | Y | 9 | 1 | 6 | Critically low |
| Li et al.  (2017) | Y | N | N | PY | N | Y | N | Y | PY | N | Y | Y | Y | Y | Y | Y | 9 | 2 | 5 | Critically low |
| Chen et al.  (2016) | Y | N | N | Y | N | Y | N | Y | Y | N | N | N | Y | N | Y | Y | 8 | 0 | 8 | Critically low |
| Ma  (2015) | Y | N | N | PY | N | N | N | Y | Y | N | Y | Y | Y | Y | Y | N | 8 | 1 | 7 | Critically low |
| Wang  (2013) | Y | N | N | PY | Y | N | N | Y | Y | N | Y | N | N | Y | Y | N | 7 | 1 | 8 | Critically low |
| Zhang et al.  (2012) | Y | N | N | PY | Y | Y | N | Y | PY | N | Y | N | Y | Y | N | Y | 8 | 2 | 6 | Critically low |
| Ding et al.  (2011) | Y | N | N | PY | N | Y | N | PY | PY | N | Y | N | Y | Y | N | Y | 6 | 3 | 7 | Critically low |
| Lei  (2010) | Y | N | N | Y | Y | Y | N | Y | PY | N | Y | N | N | Y | Y | N | 8 | 1 | 7 | Critically low |
| Ma and Li  (2010) | N | N | N | Y | N | N | N | N | PY | N | N | N | Y | Y | Y | Y | 5 | 1 | 10 | Critically low |
| Wu et al.  (2010) | Y | N | N | PY | Y | Y | N | PY | N | N | Y | N | N | N | Y | N | 5 | 2 | 9 | Critically low |
| Su et al.  (2010) | Y | N | N | PY | N | Y | N | Y | PY | N | Y | N | Y | Y | N | N | 6 | 2 | 8 | Critically low |
| Li and Miao  (2007) | N | N | N | PY | N | N | N | Y | PY | N | Y | N | Y | Y | N | Y | 5 | 2 | 9 | Critically low |
| Liu et al.  (2006) | N | N | N | Y | Y | Y | N | PY | PY | N | Y | N | N | Y | N | N | 5 | 2 | 9 | Critically low |
| Y | 13  (81.2%) | 0  (0%) | 0  (0%) | 4  (25%) | 9  (56.2%) | 10  (62.5%) | 0  (0%) | 10  (62.5%) | 7  (43.8%) | 1  (6.2%) | 14  (87.5%) | 2  (12.5%) | 11  (68.8%) | 14  (87.5%) | 11  (68.8%) | 10  (62.5%) |  |  |  |  |
| PY | 0  (0%) | 0  (0%) | 0  (0%) | 12  (75%) | 0  (0%) | 0  (0%) | 0  (0%) | 5  (31.2%) | 8  (50%) | 0  (0%) | 0  (0%) | 0  (0%) | 0  (0%) | 0  (0%) | 0  (0%) | 0  (0%) |  |  |  |  |
| N | 3  (18.8%) | 16  (100%) | 16  (100%) | 0  (0%) | 7  (43.8%) | 6  (37.5%) | 16  (100%) | 1  (6.2%) | 1  (6.2%) | 15  (93.8%) | 2  (12.5%) | 14  (87.5%) | 5  (31.2%) | 2  (12.5%) | 5  (31.2%) | 6  (37.5%) |  |  |  |  |

Table 3 Quality of reporting of included systematic reviews assessed using the PRISMA 2020 statement.

| Item | Zhang et al.  (2024) | Li et al.  (2023) | | Zhao et al.  (2022) | | Xiang et al.  (2017) | | Li et al.  (2017) | | Chen et al.  (2016) | | Ma (2015) | | Wang (2013) | | Zhang et al.  (2012) | | Ding et al.  (2011) | | Lei (2010) | | Ma and Li  (2010) | | Wu et al.  (2010) | | Su et al.  (2010) | | Li and Miao  (2007) | | Liu et al.  (2006) | | Y  (%) | | PY  (%) | | N  (%) | |
| --- | --- | --- | --- | --- | --- | --- | --- | --- | --- | --- | --- | --- | --- | --- | --- | --- | --- | --- | --- | --- | --- | --- | --- | --- | --- | --- | --- | --- | --- | --- | --- | --- | --- | --- | --- | --- | --- |
| 1 | PY | Y | | PY | | Y | | Y | | PY | | PY | | PY | | PY | | Y | | Y | | Y | | PY | | Y | | Y | | Y | | 9  (56.2%) | | 7  (43.8%) | | 0  (0%) | |
| 2 | PY | PY | | PY | | PY | | PY | | PY | | PY | | PY | | PY | | PY | | PY | | PY | | PY | | PY | | PY | | PY | | 0  (0%) | | 16  (100%) | | 0  (0%) | |
| 3 | Y | Y | | Y | | Y | | Y | | Y | | Y | | Y | | Y | | Y | | Y | | Y | | Y | | Y | | Y | | Y | | 16  (100%) | | 0  (0%) | | 0  (0%) | |
| 4 | Y | Y | | Y | | Y | | Y | | Y | | Y | | Y | | Y | | Y | | Y | | Y | | Y | | Y | | Y | | Y | | 16  (100%) | | 0  (0%) | | 0  (0%) | |
| 5 | PY | PY | | PY | | PY | | PY | | PY | | PY | | PY | | PY | | PY | | PY | | PY | | PY | | PY | | PY | | PY | | 0  (0%) | | 16  (100%) | | 0  (0%) | |
| 6 | PY | PY | | PY | | PY | | PY | | PY | | PY | | PY | | PY | | PY | | PY | | PY | | PY | | PY | | PY | | PY | | 0  (0%) | | 16  (100%) | | 0  (0%) | |
| 7 | PY | PY | | PY | | PY | | PY | | PY | | PY | | PY | | PY | | PY | | PY | | PY | | PY | | PY | | PY | | PY | | 0  (0%) | | 16  (100%) | | 0  (0%) | |
| 8 | Y | Y | | Y | | Y | | N | | Y | | N | | Y | | Y | | N | | Y | | N | | Y | | N | | N | | Y | | 10  (62.5%) | | 0  (0%) | | 6  (37.5%) | |
| 9 | Y | Y | | Y | | N | | Y | | Y | | N | | N | | Y | | Y | | N | | N | | Y | | Y | | N | | Y | | 10  (62.5%) | | 0  (0%) | | 6  (37.5%) | |
| 10a | PY | PY | | PY | | PY | | PY | | PY | | PY | | PY | | PY | | PY | | PY | | PY | | PY | | PY | | PY | | PY | | 0  (0%) | | 16  (100%) | | 0  (0%) | |
| 10b | PY | PY | | PY | | PY | | PY | | PY | | PY | | PY | | PY | | PY | | PY | | PY | | PY | | PY | | PY | | PY | | 0  (0%) | | 16  (100%) | | 0  (0%) | |
| 11 | PY | PY | | PY | | PY | | PY | | Y | | PY | | PY | | Y | | PY | | PY | | N | | PY | | PY | | PY | | PY | | 2  (12.5%) | | 13  (81.2%) | | 1  (6.3%) | |
| 12 | Y | Y | | Y | | Y | | Y | | Y | | Y | | Y | | Y | | Y | | Y | | Y | | Y | | Y | | Y | | Y | | 16  (100%) | | 0  (0%) | | 0  (0%) | |
| 13a | Y | Y | | Y | | Y | | Y | | Y | | Y | | Y | | Y | | Y | | Y | | Y | | Y | | Y | | Y | | Y | | 16  (100%) | | 0  (0%) | | 0  (0%) | |
| 13b | Y | Y | | Y | | Y | | Y | | Y | | Y | | Y | | Y | | Y | | Y | | Y | | Y | | Y | | Y | | Y | | 16  (100%) | | 0  (0%) | | 0  (0%) | |
| 13c | Y | Y | | Y | | Y | | Y | | Y | | Y | | Y | | Y | | Y | | Y | | Y | | Y | | Y | | Y | | Y | | 16  (100%) | | 0  (0%) | | 0  (0%) | |
| 13d | Y | Y | | Y | | Y | | Y | | Y | | Y | | Y | | Y | | Y | | Y | | Y | | Y | | Y | | Y | | Y | | 16  (100%) | | 0  (0%) | | 0  (0%) | |
| 13e | Y | N | | Y | | Y | | Y | | N | | Y | | Y | | N | | Y | | Y | | N | | N | | Y | | N | | Y | | 10  (62.5%) | | 0  (0%) | | 6  (37.5%) | |
| 13f | N | N | | Y | | Y | | N | | N | | Y | | N | | N | | Y | | Y | | Y | | Y | | N | | Y | | Y | | 9  (56.2%) | | 0  (0%) | | 7  (43.8%) | |
| 14 | Y | Y | | Y | | Y | | Y | | Y | | Y | | Y | | Y | | Y | | Y | | Y | | Y | | Y | | Y | | Y | | 16  (100%) | | 0  (0%) | | 0  (0%) | |
| 15 | N | Y | | Y | | Y | | N | | N | | N | | Y | | N | | N | | N | | N | | N | | N | | N | | N | | 4  (25%) | | 0  (0%) | | 12  (75%) | |
| 16a | Y | Y | | Y | | Y | | Y | | Y | | Y | | Y | | Y | | Y | | Y | | Y | | PY | | PY | | Y | | Y | | 14  (87.5%) | | 2  (12.5%) | | 0  (0%) | |
| 16b | N | N | | N | | N | | N | | N | | N | | N | | N | | N | | N | | N | | N | | N | | N | | N | | 0  (0%) | | 0  (0%) | | 16  (100%) | |
| 17 | Y | Y | | Y | | Y | | Y | | Y | | Y | | Y | | Y | | Y | | Y | | PY | | Y | | Y | | PY | | Y | | 14  (87.5%) | | 2  (12.5%) | | 0  (0%) | |
| 18 | Y | Y | | Y | | Y | | Y | | Y | | Y | | Y | | PY | | Y | | Y | | PY | | PY | | Y | | Y | | Y | | 13  (81.2%) | | 3  (18.8%) | | 0  (0%) | |
| 19 | Y | Y | | Y | | Y | | Y | | Y | | Y | | Y | | Y | | Y | | Y | | Y | | Y | | Y | | Y | | Y | | 16  (100%) | | 0  (0%) | | 0  (0%) | |
| 20a | PY | PY | | PY | | PY | | PY | | PY | | PY | | PY | | PY | | PY | | PY | | PY | | PY | | PY | | PY | | PY | | 0  (0%) | | 16  (100%) | | 0  (0%) | |
| 20b | Y | Y | | Y | | Y | | Y | | Y | | Y | | Y | | Y | | Y | | Y | | Y | | Y | | Y | | Y | | Y | | 16  (100%) | | 0  (0%) | | 0  (0%) | |
| 20c | Y | N | | Y | | Y | | Y | | N | | Y | | Y | | Y | | Y | | N | | N | | N | | Y | | Y | | N | | 8  (50%) | | 0  (0%) | | 8  (50%) | |
| 20d | Y | Y | | Y | | Y | | Y | | N | | Y | | N | | N | | Y | | N | | Y | | Y | | N | | Y | | N | | 10  (62.5%) | | 0  (0%) | | 6  (37.5%) | |
| 21 | Y | Y | | PY | | PY | | N | | PY | | Y | | PY | | N | | N | | N | | N | | N | | N | | N | | N | | 3  (18.8%) | | 4  (25%) | | 9  (56.2%) | |
| 22 | N | Y | | Y | | Y | | N | | N | | N | | Y | | N | | N | | N | | N | | N | | N | | N | | N | | 4  (25%) | | 0  (0%) | | 12  (75%) | |
| 23a | Y | Y | | Y | | Y | | Y | | Y | | Y | | Y | | Y | | Y | | Y | | Y | | Y | | Y | | Y | | Y | | 16  (100%) | | 0  (0%) | | 0  (0%) | |
| 23b | Y | Y | | Y | | Y | | Y | | Y | | Y | | Y | | Y | | Y | | Y | | Y | | Y | | Y | | Y | | Y | | 16  (100%) | | 0  (0%) | | 0  (0%) | |
| 23c | Y | Y | | Y | | Y | | Y | | Y | | Y | | Y | | Y | | Y | | Y | | Y | | Y | | Y | | Y | | Y | | 16  (100%) | | 0  (0%) | | 0  (0%) | |
| 23d | Y | Y | | Y | | Y | | Y | | Y | | Y | | Y | | Y | | Y | | Y | | Y | | Y | | Y | | Y | | Y | | 16  (100%) | | 0  (0%) | | 0  (0%) | |
| 24a | N | N | | N | | N | | N | | N | | N | | N | | N | | N | | N | | N | | N | | N | | N | | N | | 0  (0%) | | 0  (0%) | | 16  (100%) | |
| 24b | N | N | | N | | N | | N | | N | | N | | N | | N | | N | | N | | N | | N | | N | | N | | N | | 0  (0%) | | 0  (0%) | | 16  (100%) | |
| 24c | N | N | | N | | N | | N | | N | | N | | N | | N | | N | | N | | N | | N | | N | | N | | N | | 0  (0%) | | 0  (0%) | | 16  (100%) | |
| 25 | Y | Y | | Y | | Y | | Y | | Y | | Y | | Y | | Y | | Y | | Y | | Y | | N | | N | | Y | | N | | 13  (81.2%) | | 0  (0%) | | 3  (18.8%) | |
| 26 | N | N | | N | | N | | N | | N | | N | | N | | N | | N | | N | | Y | | N | | N | | Y | | N | | 2  (12.5%) | | 0  (0%) | | 14  (87.5%) | |
| 27 | N | N | | N | | N | | N | | N | | N | | N | | N | | N | | N | | N | | N | | N | | N | | N | | 0  (0%) | | 0  (0%) | | 16  (100%) | |
| N | 9 | | 9 | | 6 | | 7 | | 11 | | 12 | | 10 | | 9 | | 12 | | 10 | | 12 | | 13 | | 12 | | 13 | | 11 | | 12 | |  | |  | |  |
| PY | 9 | | 8 | | 10 | | 9 | | 8 | | 9 | | 9 | | 10 | | 9 | | 8 | | 8 | | 9 | | 11 | | 9 | | 9 | | 8 | |  | |  | |  |
| Y | 24 | | 25 | | 26 | | 26 | | 23 | | 21 | | 23 | | 23 | | 21 | | 24 | | 22 | | 20 | | 19 | | 20 | | 22 | | 22 | |  | |  | |  |
| Full Score | 28.5 | | 29 | | 31 | | 30.5 | | 27 | | 25.5 | | 27.5 | | 28 | | 25.5 | | 28 | | 26 | | 24.5 | | 24.5 | | 24.5 | | 26.5 | | 26 | |  | |  | |  |
| Quality Level | Moderate | | Moderate | | Moderate | | Moderate | | Moderate | | Moderate | | Moderate | | Moderate | | Moderate | | Moderate | | Moderate | | Low | | Low | | Low | | Moderate | | Moderate | |  | |  | |  |

Table 4 Risk of bias of included SRs with ROBIS tool

| Review (Year) | Phase 1 | Phase 2 | | | | Phase 3 |
| --- | --- | --- | --- | --- | --- | --- |
|  |  | Domain 1 | Domain 2 | Domain 3 | Domain 4 |  |
| Zhang et al. (2024) | Low | Low | High | Low | High | High |
| Li et al. (2023) | Low | Low | High | Low | High | High |
| Zhao et al. (2022) | Low | Low | High | High | High | High |
| Xiang et al. (2017) | Low | Low | High | High | High | High |
| Li et al. (2017) | Low | Low | High | High | High | High |
| Chen et al. (2016) | Low | Low | High | Low | High | High |
| Ma (2015) | Low | Low | High | High | High | High |
| Wang (2013) | Low | Low | High | High | High | High |
| Zhang et al. (2012) | Low | Low | High | High | High | High |
| Ding et al. (2011) | Low | Low | Unclear | Low | High | High |
| Lei (2010) | Low | High | Unclear | Low | High | High |
| Ma and Li (2010) | Low | High | High | Unclear | High | High |
| Wu et al. (2010) | Low | High | High | Unclear | High | High |
| Su et al. (2010) | Low | Low | High | High | High | High |
| Li and Miao (2007) | Low | High | High | Unclear | High | High |
| Liu et al. (2006) | Low | High | Unclear | Low | High | High |

Table 5 Risk of bias of included SRs with ROBIS tool (Phase 2 and phase 3)

| Item | | | Zhang et al.  (2024) | Li et al.  (2023) | Zhao et al.  (2022) | Xiang et al.  (2017) | Li et al.  (2017) | Chen et al.  (2016) | Ma (2015) | Wang (2013) | Zhang et al.  (2012) | Ding et al.  (2011) | Lei (2010) | Ma and Li  (2010) | Wu et al.  (2010) | Su et al.  (2010) | Li and Miao  (2007) | Liu et al.  (2006) |
| --- | --- | --- | --- | --- | --- | --- | --- | --- | --- | --- | --- | --- | --- | --- | --- | --- | --- | --- |
| Phase 2 | Domain 1 | 1.1 | PY | PY | PY | PY | PY | PY | PY | PY | PY | PY | PN | PN | PN | PY | PN | PY |
|  |  | 1.2 | Y | Y | Y | Y | Y | Y | Y | Y | Y | Y | Y | Y | Y | Y | Y | Y |
|  |  | 1.3 | Y | Y | Y | Y | Y | Y | Y | Y | Y | Y | Y | Y | PN | Y | Y | Y |
|  |  | 1.4 | Y | Y | Y | Y | Y | Y | PY | PY | Y | Y | PN | PY | Y | PY | PY | PN |
|  |  | 1.5 | Y | Y | Y | Y | Y | Y | Y | Y | Y | Y | Y | Y | Y | Y | Y | Y |
|  | Domain 2 | 2.1 | N | N | N | N | N | Y | N | Y | N | N | Y | Y | N | N | Y | Y |
|  |  | 2.2 | N | N | N | N | N | Y | N | N | N | N | Y | Y | N | N | Y | Y |
|  |  | 2.3 | Y | N | Y | N | Y | N | N | N | Y | N | Y | N | N | N | N | N |
|  |  | 2.4 | Y | Y | Y | Y | Y | Y | Y | Y | Y | Y | Y | Y | Y | Y | N | Y |
|  |  | 2.5 | Y | Y | Y | Y | N | N | N | Y | Y | NI | NI | N | N | N | N | NI |
|  | Domain 3 | 3.1 | Y | Y | N | N | Y | Y | N | N | Y | Y | Y | NI | Y | Y | NI | Y |
|  |  | 3.2 | Y | Y | Y | Y | Y | Y | Y | Y | Y | Y | Y | PN | Y | Y | PN | Y |
|  |  | 3.3 | Y | Y | Y | Y | Y | Y | Y | Y | Y | Y | Y | Y | Y | Y | Y | Y |
|  |  | 3.4 | Y | Y | Y | Y | Y | Y | Y | Y | Y | Y | Y | Y | NI | Y | Y | Y |
|  |  | 3.5 | Y | Y | N | Y | N | Y | N | N | Y | N | Y | N | NI | N | N | Y |
|  | Domain 4 | 4.1 | PN | PN | PN | PN | PN | PN | PN | PN | PN | PN | PN | PN | PN | PN | PN | PN |
|  |  | 4.2 | NI | NI | NI | NI | NI | NI | NI | NI | NI | NI | NI | NI | NI | NI | NI | NI |
|  |  | 4.3 | Y | Y | Y | Y | Y | Y | Y | Y | Y | Y | Y | Y | Y | Y | Y | Y |
|  |  | 4.4 | Y | Y | Y | Y | Y | Y | Y | Y | Y | Y | Y | Y | Y | Y | Y | Y |
|  |  | 4.5 | Y | Y | Y | Y | Y | N | Y | N | N | N | Y | Y | Y | N | Y | N |
|  |  | 4.6 | N | N | N | N | N | N | N | N | N | N | N | N | N | N | N | N |
| Phase 3 | | A | N | N | N | N | N | N | N | N | N | N | N | N | N | N | N | N |
|  |  | B | Y | Y | Y | Y | Y | Y | Y | Y | Y | Y | Y | Y | Y | Y | Y | Y |
|  |  | C | Y | Y | Y | Y | Y | Y | Y | Y | Y | Y | Y | Y | Y | Y | Y | Y |

Table 6 Qualities of the evidence measuring outcomes rated by the GRADE system

| Review (Year) | Outcome | Intervention Measure | | Number of Primary Studies (Sample Size) | Risk of Bias | Inconsistency | Indirectness | Imprecision | Publication Bias | Grade | Quality |
| --- | --- | --- | --- | --- | --- | --- | --- | --- | --- | --- | --- |
|  |  | Treatment Group | Control Group |  |  |  |  |  |  |  |  |
| Zhang et al.  (2024) | Total effective rate | SXT+CT | CT | 6(539) | -1 | 0 | 0 | -1 | 0 | -2 | Low |
|  | NIHSS | SXT+CT | CT | 7(657) | -1 | -1 | 0 | 0 | 0 | -2 | Low |
|  | BI | SXT+CT | CT | 3(278) | -1 | 0 | 0 | -1 | 0 | -2 | Low |
|  | HSV | SXT+CT | CT | 3(278) | -1 | 0 | 0 | -1 | 0 | -2 | Low |
|  | LSV | SXT+CT | CT | 3(278) | -1 | 0 | 0 | -1 | 0 | -2 | Low |
|  | PV | SXT+CT | CT | 2(202) | -1 | -1 | 0 | -1 | 0 | -3 | Very Low |
|  | Fb | SXT+CT | CT | 2(180) | -1 | -1 | 0 | -1 | 0 | -3 | Very Low |
|  | CRP | SXT+CT | CT | 2(159) | -1 | 0 | 0 | -1 | 0 | -2 | Low |
|  | TNF-α | SXT+CT | CT | 2(159) | -1 | -1 | 0 | -2 | 0 | -4 | Very Low |
| Li et al.  (2023) | Total effective rate | SXT+CT | CT | 26(2382) | -1 | 0 | 0 | 0 | -1 | -2 | Low |
|  | NIHSS | SXT+CT | CT | 19(1673) | -1 | -1 | 0 | 0 | -1 | -3 | Very Low |
|  | BI | SXT+CT | CT | 5(336) | -1 | 0 | 0 | -2 | -1 | -4 | Very Low |
|  | WBV | SXT+CT | CT | 4(346) | -1 | -1 | 0 | -1 | -1 | -4 | Very Low |
|  | PV | SXT+CT | CT | 8(657) | -1 | -1 | 0 | 0 | -1 | -3 | Low |
|  | Fb | SXT+CT | CT | 7(608) | -1 | -1 | 0 | 0 | -1 | -3 | Low |
|  | hs-CRP | SXT+CT | CT | 8(858) | -1 | -1 | 0 | 0 | -1 | -3 | Low |
|  | TNF-α | SXT+CT | CT | 3(282) | -1 | 0 | 0 | -1 | -1 | -3 | Low |
| Zhao et al.  (2022) | Total effective rate | SXT+CT | CT | 17(1512) | -1 | 0 | 0 | 0 | -1 | -2 | Low |
|  | Deterioration rate | SXT+CT | CT | 8(772) | -1 | 0 | 0 | 0 | 0 | -1 | Moderate |
|  | Mortality rate | SXT+CT | CT | 6(668) | -1 | 0 | 0 | 0 | 0 | -1 | Moderate |
|  | NIHSS | SXT+CT | CT | 5(392) | -1 | 0 | 0 | -1 | 0 | -2 | Low |
|  | CSS | SXT+CT | CT | 10(1042) | -1 | 0 | 0 | 0 | 0 | -1 | Moderate |
|  | ADL | SXT+CT | CT | 5(426) | -1 | 0 | 0 | -2 | 0 | -3 | Very Low |
|  | PT | SXT+CT | CT | 5(436) | -1 | -1 | 0 | -2 | 0 | -4 | Very Low |
|  | APTT | SXT+CT | CT | 3(288) | -1 | -1 | 0 | -1 | 0 | -3 | Very Low |
|  | TT | SXT+CT | CT | 2(162) | -1 | -1 | 0 | -2 | 0 | -4 | Very Low |
|  | Fb | SXT+CT | CT | 7(564) | -1 | -1 | 0 | 0 | 0 | -2 | Low |
|  | TC | SXT+CT | CT | 2(174) | -1 | -1 | 0 | -2 | 0 | -4 | Very Low |
|  | TG | SXT+CT | CT | 2(174) | -1 | -1 | 0 | -1 | 0 | -3 | Very Low |
|  | LDL-c | SXT+CT | CT | 2(174) | -1 | 0 | 0 | -1 | 0 | -2 | Low |
|  | hs-CRP | SXT+CT | CT | 2(166) | -1 | -1 | 0 | -2 | 0 | -4 | Very Low |
|  | IL-6 | SXT+CT | CT | 2(166) | -1 | 0 | 0 | -2 | 0 | -3 | Very Low |
| Xiang et al.  (2017) | Total Effective rate | SXT+CT | CT | 6(312) | -1 | 0 | 0 | -1 | -1 | -3 | Very Low |
|  |  | SXT+CT | CT | 2(99) | -1 | 0 | 0 | -1 | -1 | -3 | Very Low |
|  | NIHSS | SXT+CT | CT | 3(384) | -1 | -1 | 0 | -2 | -1 | -5 | Very Low |
|  | CSS | SXT+CT | CT | 2(224) | -1 | -1 | 0 | -2 | -1 | -5 | Very Low |
|  | PV | SXT+CT | CT | 8(822) | -1 | -1 | 0 | 0 | -1 | -3 | Very Low |
|  | HSV | SXT+CT | CT | 5(582) | -1 | -1 | 0 | -1 | -1 | -4 | Very Low |
|  | LSV | SXT+CT | CT | 5(582) | -1 | -1 | 0 | -2 | -1 | -5 | Very Low |
|  | WBV | SXT+CT | CT | 3(240) | -1 | -1 | 0 | -1 | -1 | -4 | Very Low |
|  | FIB | SXT+CT | CT | 5(518) | -1 | -1 | 0 | -1 | -1 | -4 | Very Low |
|  | ESR | SXT+CT | CT | 2(198) | -1 | 0 | 0 | -2 | -1 | -4 | Very Low |
|  | EAI | SXT+CT | CT | 3(336） | -1 | -1 | 0 | -2 | -1 | -5 | Very Low |
|  | HCT | SXT+CT | CT | 2(240) | -1 | -1 | 0 | -2 | -1 | -5 | Very Low |
| Li et al.  (2017) | Total effective rate | SXT+CT | Ginkgo Damo+CT | 3(329) | -1 | 0 | 0 | -1 | 0 | -2 | Low |
|  |  | SXT+CT | Shuxuening+CT | 7(909) | -1 | 0 | 0 | 0 | 0 | -1 | Moderate |
|  | Neurological deficit score | SXT+CT | Ginkgo Damo+CT | 3(317) | -1 | -1 | 0 | -2 | 0 | -4 | Very Low |
|  |  | SXT+CT | Shuxuening+CT | 7(909) | -1 | -1 | 0 | -1 | 0 | -3 | Very Low |
|  | ADL | SXT+CT | Ginkgo Damo+CT | 4(429) | -1 | 0 | 0 | -2 | 0 | -3 | Very Low |
| Chen et al.  (2016) | Total effective rate | SXT+CT | Notoginseng+CT | 13(1242) | -1 | 0 | 0 | 0 | 0 | -1 | Moderate |
|  | CSS | SXT+CT | Notoginseng+CT | 13(1242) | -1 | -2 | 0 | 0 | 0 | -3 | Very Low |
|  | ADL | SXT+CT | Notoginseng+CT | 3(314) | -1 | -2 | 0 | -1 | 0 | -4 | Very Low |
| Ma (2015) | Total effective rate | SXT+CT | Xuesaitong+CT | 6(572) | -1 | 0 | 0 | 0 | 0 | -1 | Moderate |
|  | CSS | SXT+CT | Xuesaitong+CT | 5(510) | -1 | -1 | 0 | -2 | 0 | -4 | Very Low |
|  | HSV | SXT+CT | Xuesaitong+CT | 2(146) | -1 | -1 | 0 | -1 | 0 | -3 | Very Low |
|  | LSV | SXT+CT | Xuesaitong+CT | 2(146) | -1 | -1 | 0 | -2 | 0 | -4 | Very Low |
|  | Fb | SXT+CT | Xuesaitong+CT | 3(208) | -1 | -1 | 0 | -1 | 0 | -3 | Very Low |
|  | HCT | SXT+CT | Xuesaitong+CT | 2(146) | -1 | -1 | 0 | -1 | 0 | -3 | Very Low |
| Wang et al.  (2013) | Total effective rate | SXT+CT | Other Injections+CT | 18(1962) | -1 | 0 | 0 | 0 | 0 | -1 | Moderate |
|  | Neurological deficit score | SXT+CT | Other Injections+CT | 10(957) | -1 | -1 | 0 | -1 | 0 | -3 | Very Low |
|  | HSV | SXT+CT | Other Injections+CT | 7(575) | -1 | -1 | 0 | 0 | 0 | -2 | Low |
|  | LSV | SXT+CT | Other Injections+CT | 4(363) | -1 | -1 | 0 | -2 | 0 | -4 | Very Low |
|  | PV | SXT+CT | Other Injections+CT | 8(727) | -1 | -1 | 0 | 0 | 0 | -2 | Low |
|  | Packed cell volume | SXT+CT | Other Injections+CT | 7(615) | -1 | -1 | 0 | -1 | 0 | -3 | Very Low |
|  | EAI | SXT+CT | Other Injections+CT | 3(303) | -1 | -1 | 0 | -1 | 0 | -3 | Very Low |
|  | FIB | SXT+CT | Other Injections+CT | 8(705) | -1 | -1 | 0 | 0 | 0 | -2 | Low |
| Zhang et al.  (2012) | Total Effective rate | SXT+CT | CT | 11(972) | -1 | 0 | 0 | -1 | -2 | -4 | Very Low |
|  | CSS | SXT+CT | CT | 7(740) | -1 | 0 | 0 | -1 | -2 | -4 | Very Low |
| Ding et al.  (2011) | Total Effective rate | SXT+CT | Other Injections+CT | 10(938) | -1 | 0 | 0 | 0 | -2 | -3 | Very Low |
|  | Efficacy rate | SXT+CT | Other Injections+CT | 2(179) | -1 | 0 | 0 | -1 | -2 | -4 | Very Low |
|  | CSS | SXT+CT | Other Injections+CT | 3(312) | -1 | 0 | 0 | -2 | -2 | -5 | Very Low |
|  |  | SXT+CT | Other Injections+CT | 1(60) | -1 | 0 | 0 | -2 | -2 | -5 | Very Low |
|  |  | SXT+CT | Other Injections+CT | 1(63) | -1 | 0 | 0 | -2 | -2 | -5 | Very Low |
| Lei et al.  (2010) | Efficacy rate | SXT+CT | Other Injections+CT | 12(1210) | -1 | 0 | 0 | 0 | -1 | -2 | Low |
|  | NIHSS | SXT+CT | Other Injections+CT | 1(78) | -1 | 0 | 0 | -1 | -1 | -3 | Very Low |
|  | CSS | SXT+CT | Other Injections+CT | 7(665) | -1 | -1 | 0 | 0 | -1 | -3 | Very Low |
|  | HSV | SXT+CT | Other Injections+CT | 4(332) | -1 | -1 | 0 | -1 | -1 | -4 | Very Low |
|  | LSV | SXT+CT | Other Injections+CT | 4(332) | -1 | 0 | 0 | -1 | -1 | -3 | Very Low |
|  | WBV | SXT+CT | Other Injections+CT | 2(318) | -1 | 0 | 0 | -1 | -1 | -3 | Very Low |
|  | PV | SXT+CT | Other Injections+CT | 7(736) | -1 | -1 | 0 | 0 | -1 | -3 | Very Low |
|  | FIB | SXT+CT | Other Injections+CT | 7(736) | -1 | -1 | 0 | 0 | -1 | -3 | Very Low |
|  | Packed cell volume | SXT+CT | Other Injections+CT | 7(736) | -1 | -1 | 0 | 0 | -1 | -3 | Very Low |
|  | Platelet adhesion rate | SXT+CT | Other Injections+CT | 3(286) | -1 | -1 | 0 | -1 | -1 | -4 | Very Low |
| Ma and Li  (2010) | Total Effective rate | SXT+CT | CT | 23(2066) | -2 | 0 | 0 | 0 | -1 | -3 | Very Low |
|  | CSS | SXT+CT | CT | 12(1125) | -2 | 0 | 0 | -1 | -1 | -4 | Very Low |
| Wu et al.  (2010) | Efficacy rate | SXT+CT | Other Injections+CT | 28(2718) | -2 | 0 | 0 | 0 | 0 | -2 | Low |
|  | CSS | SXT+CT | Other Injections+CT | 13(1337) | -2 | -2 | 0 | -1 | 0 | -5 | Very Low |
| Su et al.  (2012) | Recovery Rate | SXT+CT | CT | 6(447) | -1 | 0 | -1 | 0 | -2 | -4 | Very Low |
|  |  | SXT+CT | Edaravone+CT | 4(466) | -1 | 0 | 0 | -1 | -2 | -4 | Very Low |
|  | Total Effective rate | SXT+CT | CT | 6(447) | -1 | 0 | -1 | 0 | -2 | -4 | Very Low |
|  |  | SXT+CT | Edaravone+CT | 4(466) | -1 | 0 | 0 | -1 | -2 | -4 | Very Low |
| Li and Miao  (2007) | Total effective rate | SXT+CT | CT | 3(315) | -2 | 0 | 0 | -1 | -2 | -5 | Very Low |
|  | CSS | SXT+CT | CT | 3(315) | -2 | -1 | 0 | -2 | -2 | -7 | Very Low |
|  | BI | SXT+CT | CT | 1(78) | -2 | 0 | 0 | -2 | -2 | -6 | Very Low |
| Liu et al.  (2006) | Total effective rate | SXT+CT | Other Injections+CT | 11(1122) | -1 | 0 | 0 | -1 | -2 | -4 | Very Low |
|  | CSS | SXT+CT | Other Injections+CT | 1（63） | -1 | 0 | 0 | -1 | -2 | -4 | Very Low |
|  |  | SXT+CT | Other Injections+CT | 1（115） | -1 | 0 | 0 | -1 | -2 | -4 | Very Low |
|  |  | SXT+CT | Other Injections+CT | 1（92） | -1 | 0 | 0 | -1 | -2 | -4 | Very Low |
|  | HSV | SXT+CT | Other Injections+CT | 1(87) | -1 | 0 | 0 | -1 | -2 | -4 | Very Low |
|  |  | SXT+CT | Other Injections+CT | 1(105) | -1 | 0 | 0 | -1 | -2 | -4 | Very Low |
|  |  | SXT+CT | Other Injections+CT | 1(92) | -1 | 0 | 0 | -1 | -2 | -4 | Very Low |
|  |  | SXT+CT | Other Injections+CT | 1(120) | -1 | 0 | 0 | -1 | -2 | -4 | Very Low |
|  | LSV | SXT+CT | Other Injections+CT | 1(87) | -1 | 0 | 0 | -1 | -2 | -4 | Very Low |
|  |  | SXT+CT | Other Injections+CT | 1(105) | -1 | 0 | 0 | -1 | -2 | -4 | Very Low |
|  |  | SXT+CT | Other Injections+CT | 1(120) | -1 | 0 | 0 | -1 | -2 | -4 | Very Low |
|  | PV | SXT+CT | Other Injections+CT | 1(87) | -1 | 0 | 0 | -1 | -2 | -4 | Very Low |
|  |  | SXT+CT | Other Injections+CT | 1(105) | -1 | 0 | 0 | -1 | -2 | -4 | Very Low |
|  |  | SXT+CT | Other Injections+CT | 1(92) | -1 | 0 | 0 | -1 | -2 | -4 | Very Low |
|  |  | SXT+CT | Other Injections+CT | 1(100) | -1 | 0 | 0 | -1 | -2 | -4 | Very Low |
|  |  | SXT+CT | Other Injections+CT | 1(120) | -1 | 0 | 0 | -1 | -2 | -4 | Very Low |
|  | HCT | SXT+CT | Other Injections+CT | 1(87) | -1 | 0 | 0 | -2 | -2 | -5 | Very Low |
|  |  | SXT+CT | Other Injections+CT | 1(105) | -1 | 0 | 0 | -2 | -2 | -5 | Very Low |
|  |  | SXT+CT | Other Injections+CT | 1(100) | -1 | 0 | 0 | -2 | -2 | -5 | Very Low |
|  | FIB | SXT+CT | Other Injections+CT | 1(87) | -1 | 0 | 0 | -1 | -2 | -4 | Very Low |
|  |  | SXT+CT | Other Injections+CT | 1(105) | -1 | 0 | 0 | -1 | -2 | -4 | Very Low |
|  |  | SXT+CT | Other Injections+CT | 1(100) | -1 | 0 | 0 | -1 | -2 | -4 | Very Low |
|  |  | SXT+CT | Other Injections+CT | 1(120) | -1 | 0 | 0 | -1 | -2 | -4 | Very Low |
|  | EAI | SXT+CT | Other Injections+CT | 1(105) | -1 | 0 | 0 | -1 | -2 | -4 | Very Low |
|  | Platelet adhesion rate | SXT+CT | Other Injections+CT | 1(92) | -1 | 0 | 0 | -2 | -2 | -5 | Very Low |
|  | TNF-α | SXT+CT | Other Injections+CT | 1(115) | -1 | 0 | 0 | -1 | -2 | -4 | Very Low |
|  | sICAM-1 | SXT+CT | Other Injections+CT | 1(115) | -1 | 0 | 0 | -2 | -2 | -5 | Very Low |
|  | TG | SXT+CT | Other Injections+CT | 1(152) | -1 | 0 | 0 | -1 | -2 | -4 | Very Low |
|  | TC | SXT+CT | Other Injections+CT | 1(152) | -1 | 0 | 0 | -1 | -2 | -4 | Very Low |
|  | HDL-C | SXT+CT | Other Injections+CT | 1(152) | -1 | 0 | 0 | -1 | -2 | -4 | Very Low |
|  | LDL-C | SXT+CT | Other Injections+CT | 1(152) | -1 | 0 | 0 | -1 | -2 | -4 | Very Low |

Table 7 Data Extraction

| Review  (Year) | Outcome | | Intervention Measure | | | Number of Primary Studie | Sample Size | Pooled Effect Size | P | I^2^ |
| --- | --- | --- | --- | --- | --- | --- | --- | --- | --- | --- |
|  |  |  | Treatment Group | | Control Group |  |  |  |  |  |
| Zhang et al.  (2024) | Total effective rate | | CT | | SXT+CT | 6 | 539 | OR=3.60, 95%CI (2.12, 6.10) | <0.00001 | 0% |
|  | NIHSS | | CT | | SXT+CT | 7 | 657 | MD=-2.24, 95%CI (-2.92, -1.55) | <0.00001 | 97% |
|  | BI | | CT | | SXT+CT | 3 | 278 | MD=10.28, 95%CI (9.94, 10.62) | <0.00001 | 0% |
|  | HSV | | CT | | SXT+CT | 3 | 278 | MD=-0.61, 95%CI (-0.74, -0.48) | <0.00001 | 0% |
|  | LSV | | CT | | SXT+CT | 3 | 278 | MD=-1.84, 95%CI (-2.06, -1.61) | <0.00001 | 0% |
|  | PV | | CT | | SXT+CT | 2 | 202 | MD=-0.14, 95%CI (-0.39, 0.12) | 0.29 |  |
|  | Fb | | CT | | SXT+CT | 2 | 180 | MD=-0.72, 95%CI (-1.40, -0.04) | 0.04 | 98% |
|  | CRP | | CT | | SXT+CT | 2 | 159 | SMD=-0.67, 95%CI (-0.99, -0.35) | <0.0001 | 0% |
|  | TNF-α | | CT | | SXT+CT | 2 | 159 | SMD=-2.22, 95%CI (-4.35, -0.09) | 0.04 | 96% |
|  | AEs | Gastrointestinal bleeding |  | |  | 1 | 3 |  |  |  |
|  |  | Mucosal bleeding |  | |  | 1 | 2 |  |  |  |
|  |  | Symptomatic intracranial hemorrhage |  | |  | 1 | 7 |  |  |  |
|  |  | Nausea |  | |  | 1 | 13 |  |  |  |
|  |  | Vomiting |  | |  | 1 | 12 |  |  |  |
| Li et al.  (2023) | NIHSS | | CT | | SXT+CT | 19 | 1673 | MD=-3.71, 95%CI (-4.42, -2.99) | <0.01 | 83% |
|  | BI | | CT | | SXT+CT | 5 | 336 | MD=16.5, 95%CI (12.96, 20.04) | <0.01 | 45% |
|  | Total effective rate | | CT | | SXT+CT | 26 | 2382 | RR=1.15, 95%CI (1.12, 1.19) | <0.01 | 0% |
|  | WBV | | CT | | SXT+CT | 4 | 346 | MD=-1.49, 95%CI (-2.06, -0.92) | <0.01 | 96% |
|  | PV | | CT | | SXT+CT | 8 | 657 | MD=-1.11, 95%CI (-1.44, -0.78) | <0.01 | 74% |
|  | Fb | | CT | | SXT+CT | 7 | 608 | MD=-0.61, 95%CI (-1.02, -0.20) | <0.01 | 96% |
|  | hs-CRP | | CT | | SXT+CT | 8 | 858 | SMD=-1.35, 95%CI (-1.85, -0.84) | <0.01 | 91% |
|  | TNF-α | | CT | | SXT+CT | 3 | 282 | SMD=-1.07, 95%CI (-1.33, -0.82) | <0.01 | 0% |
|  | AEs | | CT | | SXT+CT |  |  |  |  |  |
| Zhao et al.  (2022) | Total effective rate | | CT | | SXT+CT | 17 | 1512 | RR=1.27, 95%CI (1.20, 1.33) | <0.00001 | 19% |
|  | Deterioration rate | | CT | | SXT+CT | 8 | 772 | RR=0.38, 95% CI (0.22, 0.68) | 0.0009 | 0% |
|  | Mortality rate | | CT | | SXT+CT | 6 | 668 | RR=0.40, 95%CI (0.13, 1.26) | 0.12 | 0% |
|  | NIHSS | | CT | | SXT+CT | 5 |  | MD=-3.89, 95% CI (-4.34, -3.43) | <0.00001 | 0% |
|  | CSS | | CT | | SXT+CT | 10 |  | MD=-5.59, 95%CI (-6.42, -4.76) | <0.00001 | 47% |
|  | ADL | | CT | | SXT+CT | 5 | 426 | MD=12.02, 95%CI (10.31, 13.72) | <0.00001 | 0% |
|  | PT | | CT | | SXT+CT | 5 |  | MD=1.22, 95%CI (-0.87, 3.31) | 0.25 | 97% |
|  | APTT | | CT | | SXT+CT | 3 |  | MD=0.80, 95%CI (-3.26, 4.85) | 0.7 | 97% |
|  | TT | | CT | | SXT+CT | 2 |  | MD=3.12, 95%CI (-1.72, 7.97) | 0.21 | 94% |
|  | Fb | | CT | | SXT+CT | 7 | 564 | MD=-0.35, 95%CI (-0.58, -0.13) | 0.002 | 80% |
|  | TC | | CT | | SXT+CT | 2 |  | MD=-0.83, 95%CI (-1.94, 0.27) | 0.14 | 98% |
|  | TG | | CT | | SXT+CT | 2 |  | MD=-0.38, 95%CI (-0.67, -0.10) | 0.008 | 90% |
|  | LDL-c | | CT | | SXT+CT | 2 |  | MD=-0.72, 95%CI (-0.83, -0.61) | <0.00001 | 0% |
|  | hs-CRP | | CT | | SXT+CT | 2 |  | MD=-4.41, 95%CI (-6.96, -1.86) | 0.0007 | 84% |
|  | IL-6 | | CT | | SXT+CT | 2 |  | MD=-5.43, 95%CI (-6.91, -3.96) | <0.00001 | 0% |
|  | AEs | Dizziness |  | |  | 1 | 3 |  |  |  |
|  |  | Palpitation |  | |  | 1 | 4 |  |  |  |
|  |  | Chest tightness |  | |  | 1 | 1 |  |  |  |
|  |  | Facial flushing |  | |  | 1 | 2 |  |  |  |
| Xiang et al.  (2017) | Total effective rate | | CT | | SXT+CT | 6 | 312 | OR=3.61, 95%CI (2.25, 5.79) | <0.00001 | 0% |
|  |  |  | CT | | SXT+CT | 2 | 99 | OR=6.30, 95%CI (2.3, 17.26) | 0.0003 | 26% |
|  | NIHSS | | CT | | SXT+CT | 3 | 384 | WMD=-3.15, 95%CI (-4.66, -1.64) |  | 90% |
|  | CSS | | CT | | SXT+CT | 2 | 224 | WMD=-3.83, 95%CI (-5.27, -2.39) |  | 76% |
|  | PV | | CT | | SXT+CT | 8 | 822 | WMD=-0.12, 95%CI (-0.26, 0.02) | 0.1 | 95% |
|  | HSV | | CT | | SXT+CT | 5 | 582 | WMD=-0.61, 95%CI (-0.98, -0.24) | 0.001 | 97% |
|  | LSV | | CT | | SXT+CT | 5 | 582 | WMD=-2.03, 95%CI (-3.44, -0.62) | 0.005 | 98% |
|  | WBV | | CT | | SXT+CT | 3 | 240 | WMD=-0.60, 95%CI (-1.07, -0.12) | 0.01 | 89% |
|  | Fb | | CT | | SXT+CT | 5 | 518 | WMD=-0.53, 95%CI (-0.80, -0.26) | 0.0001 | 90% |
|  | ESR | | CT | | SXT+CT | 2 | 198 | WMD=-2.85, 95%CI (-5.67, -0.04) | 0.05 | 0% |
|  | EAI | | CT | | SXT+CT | 3 | 336 | WMD=-0.12, 95%CI (-1.28, 1.03) | 0.83 | 98% |
|  | HCT | | CT | | SXT+CT | 2 | 240 | WMD=-2.08, 95%CI (-6.14, 1.98) | 0.32 | 99% |
|  | AEs (Mild gastrointestinal discomfort) | |  | |  | 1 | 2 |  |  |  |
| Li et al.  (2017) | Total effective rate | | Ginkgo biloba extract+CT | | SXT+CT | 10 | 1238 | RR=1.17, 95%CI (1.11, 1.23) | <0.00001 | 0% |
|  |  |  | Ginkgo Damo +CT | | SXT+CT | 3 | 329 | RR=1.22, 95%CI (1.11, 1.34) | <0.0001 | 0% |
|  |  |  | Shuxuening+CT | | SXT+CT | 7 | 909 | RR=1.15, 95%CI (1.08, 1.23) | <0.0001 | 0% |
|  | Neurological deficit score | | Ginkgo biloba extract+CT | | SXT+CT | 10 | 1226 | MD=-4.46, 95%CI (-6.07, -3.25) | <0.01 | 93% |
|  |  |  | Ginkgo Damo +CT | | SXT+CT | 3 | 317 | MD=-7.52, 95%CI (-11.64, -3.39) | 0.0004 | 77% |
|  |  |  | Shuxuening+CT | | SXT+CT | 7 | 909 | MD=-3.96, 95%CI (-5.47, -2.45) | <0.00001 | 95% |
|  | ADL | | Ginkgo Damo +CT | | SXT+CT | 4 | 429 | MD=13.98, 95%CI (11.30, 16.65) | <0.00001 | 25% |
|  | AEs (Mild non-serious AEs) | |  | |  |  |  |  |  |  |
| Chen et al.  (2016) | Total effective rate | | Xueshuantong/ Xuesaitong+CT | | SXT+CT | 13 | 1242 | RR=1.20, 95%CI (1.14, 1.26) | <0.00001 | 0% |
|  | CSS | | Xueshuantong/ Xuesaitong+CT | | SXT+CT | 13 | 1242 | SMD=-0.81, 95%CI (-0.99, -0.62) | <0.00001 | 59% |
|  | ADL | | Xueshuantong/ Xuesaitong+CT | | SXT+CT | 3 | 314 | SMD=-1.63, 95%CI (-2.20, -1.06) | <0.00001 | 79% |
|  | AEs (Mild allergic reactions) | | Xuesaitong+CT | | SXT+CT | 6 | 548 | RR= 0.85, 95%CI (0.50, 1.44) | 0.54 | 0% |
| Ma (2015) | Total effective rate | | Xuesaitong+CT | | SXT+CT | 6 | 572 | RR=1.21, 95%CI (1.12, 1.30) | <0.00001 | 28% |
|  | CSS | | Xuesaitong+CT | | SXT+CT | 5 | 510 | WMD=5.50, 95%CI (2.83, 8.18) | <0.0001 | 87% |
|  | LSV | | Xuesaitong+CT | | SXT+CT | 2 | 146 | WMD=1.60, 95%CI (‐1.97, 5.16) | 0.38 | 99% |
|  | HSV | | Xuesaitong+CT | | SXT+CT | 2 | 146 | WMD=0.60, 95%CI (‐0.06, 1.26) | 0.07 | 95% |
|  | HCT | | Xuesaitong+CT | | SXT+CT | 2 | 146 | WMD=‐0.00, 95%CI (‐0.03, 0.03) | 0.84 | 79% |
|  | FIB | | Xuesaitong+CT | | SXT+CT | 3 | 208 | WMD=0.82, 95%CI (0.07, 1.58) | 0.03 | 97% |
|  | AEs (Rash) | |  | |  | 2 | 2 |  |  |  |
| Wang (2013) | Total effective rate | | Other injection+CT | | SXT+CT | 18 | 1962 | RR=1.17, 95%CI (1.13, 1.22) | <0.00001 | 0% |
|  |  |  | Compound Danshen+CT | | SXT+CT | 5 | 686 | RR=1.15, 95%CI (1.08, 1.22) | <0.0001 | 0% |
|  |  |  | Ligustrazine+CT | | SXT+CT | 4 | 383 | RR=1.20, 95%CI (1.09, 1.31) | 0.0001 | 0% |
|  |  |  | Xuesaitong+CT | | SXT+CT | 4 | 516 | RR=1.20, 95%CI (1.12, 1.29) | <0.00001 | 47% |
|  |  |  | Xueshuantong+CT | | SXT+CT | 2 | 162 | RR=1.22, 95%CI (1.07, 1.38) | 0.002 | 0% |
|  |  |  | LMWH+CT | | SXT+CT | 1 | 63 | RR=1.00, 95%CI (0.85, 1.18) | 0.97 |  |
|  |  |  | Puerarin+CT | | SXT+CT | 1 | 60 | RR=1.17, 95%CI (0.95, 1.43) | 0.14 |  |
|  |  |  | Safflower+CT | | SXT+CT | 1 | 92 | RR=1.19, 95%CI (1.04, 1.37) | 0.01 |  |
|  | Neurological deficit score | | Other injection+CT | | SXT+CT | 10 | 957 | WMD=-2.44, 95%CI (-4.22, -0.65) | 0.007 | 93% |
|  | LSV | | Compound Danshen/ Ligustrazine/ Xueshuantong+CT | | SXT+CT | 4 | 363 | WMD=-2.09, 95%CI (-3.28, -0.91) | 0.0006 | 90% |
|  | HSV | | Compound Danshen/ Ligustrazine/ Xueshuantong+CT | | SXT+CT | 7 | 575 | WMD=-0.60, 95%CI (-1.16, -0.04) | 0.04 | 97% |
|  | PV | | Compound Danshen/ Ligustrazine/ Xueshuantong/ Puerarin/ Safflower+CT | | SXT+CT | 8 | 727 | WMD=-0.23, 95%CI (-0.33, -0.13) | <0.00001 | 72% |
|  | Packed cell volume | | Compound Danshen/ Ligustrazine/ Xueshuantong/ Puerarin/ Safflower+CT | | SXT+CT | 7 | 615 | WMD=0.32, 95%CI (-3.46, 4.09) | 0.87 | 97% |
|  | EAI | | Compound Danshen/ Ligustrazine+CT | | SXT+CT | 3 | 303 | WMD=-0.35, 95%CI (-0.64, -0.05) | 0.02 | 79% |
|  | FIB | | Compound Danshen/ Ligustrazine/ Xueshuantong/ Puerarin/ Safflower+CT | | SXT+CT | 8 | 705 | WMD=-0.78, 95%CI (-1.24, -0.32) | 0.0009 | 94% |
|  | AEs | Rash |  | |  | 2 | 2 |  |  |  |
|  |  | Pruritus |  | |  | 1 | 1 |  |  |  |
|  |  | Dizziness, palpitations with limb weakness |  | |  | 1 | 2 |  |  |  |
|  |  | Transient hypofibrinogenemia with petechiae | |  |  | 2 |  |  |  |  |
| Zhang et al.  (2012) | Total effective rate | | CT | | SXT+CT | 11 | 972 | OR=4.46, 95%CI (3.02, 6.59) | <0.00001 | 0% |
|  | CSS | | CT | | SXT+CT | 7 | 740 | MD=5.86, 95%CI (4.80, 6.93) | <0.00001 | 33% |
| Ding et al.  (2011) | Total effective rate | | Ligustrazine/ LMWH/ Puerarin/Compound Danshen/ Danshen/ Xueshuantong+CT | | SXT+CT | 10 | 938 | RR=1.17, 95%CI (1.11, 1.24) | <0.00001 | 0% |
|  | CSS | | Compound Danshen+CT | | SXT+CT | 3 | 322 | MD=-4.96, 95%CI (-6.80, -3.12) | <0.00001 | 0% |
|  |  |  | Puerarin+CT | | SXT+CT | 1 | 60 | MD=-4.49, 95%CI (-7.19, -1.19) |  |  |
|  |  |  | LMWH+CT | | SXT+CT | 1 | 63 |  | 0.36 |  |
|  | Efficacy rate | | Ligustrazine/ Danshen+CT | | SXT+CT | 2 | 179 | RR=1.49, 95%CI (1.18, 1.87) | 0.0008 | 0% |
| Lei (2010) | Efficacy rate | | Compound Danshen+CT | | SXT+CT | 6 | 539 | OR=3.11, 95%CI (2.16, 4.49) | <0.00001 | 0% |
|  |  |  | Danshen+CT | | SXT+CT | 3 | 266 | OR=3.21, 95%CI (1.90, 5.43) | <0.0001 | 0% |
|  |  |  | Xuesaitong+CT | | SXT+CT | 1 | 198 | OR=2.19, 95%CI (1.23, 3.90) | 0.008 |  |
|  |  |  | Notoginseng+CT | | SXT+CT | 1 | 87 | OR=2.44, 95%CI (1.02, 5.84) | 0.05 |  |
|  |  |  | Troxerutin+CT | | SXT+CT | 1 | 120 | OR=5.00, 95%CI (2.14, 11.66) | 0.0002 |  |
|  |  |  | Other injections+CT | | SXT+CT | 12 | 1210 | OR=3.01, 95%CI (2.36, 3.84) | <0.00001 | 0% |
|  | NIHSS | | CT | | SXT+CT | 1 | 78 | WMD=-2.70, 95%CI (-3.64, -1.76) | <0.00001 | |
|  | Neurological deficit score | | Compound Danshen/ Danshen/ Troxerutin+CT | | SXT+CT | 7 | 665 | WMD=-3.61, 95%CI (-3.61, -2.70) | <0.00001 | 58% |
|  | HSV | | Compound Danshen/ Xiangdan/ Danshen/ Notoginseng+CT | | SXT+CT | 4 | 332 | WMD=-0.69, 95%CI (-0.83, -0.55) | <0.00001 | 62% |
|  | LSV | | Compound Danshen/ Xiangdan/ Danshen/ Notoginseng+CT | | SXT+CT | 4 | 332 | WMD=-1.65, 95%CI (-1.97, -1.32) | <0.00001 | 46.70% |
|  | WBV | | Compound Danshen/ Xuesaitong+CT | | SXT+CT | 2 | 318 | WMD=-0.48, 95%CI (-0.58, -0.37) | <0.00001 | 0% |
|  | PV | | Compound Danshen/ Notoginseng/ Xiangdan/ Xuesaitong/ Danshen +CT | | SXT+CT | 7 | 736 | WMD=-0.16, 95%CI (-0.18, -0.14) | <0.00001 | 82% |
|  | Fb | | Compound Danshen/ Notoginseng/Xiangdan/ Xuesaitong/Danshen+CT | | SXT+CT | 7 | 736 | WMD=-0.35, 95%CI (-0.40, -0.31) | <0.00001 | 98% |
|  | Packed cell volume | | Compound danshen/ Notoginseng/Xiangdan/ Xuesaitong/Danshen+CT | | SXT+CT | 7 | 736 | WMD=-2.52, 95%CI (-2.94, -2.11) | <0.00001 | 98% |
|  | Platelet adhesion rate | | Compound danshen/Xiangdan/ Danshen+CT | | SXT+CT | 3 | 286 | WMD=-0.29, 95%CI (-0.51, -0.07) | 0.009 | 97% |
|  | AE | Subcutaneous ecchymosis |  | |  | 1 | 3 |  |  |  |
| Ma and Li  (2010) | Total effective rate | | CT | | SXT+CT | 23 | 2066 | RR=1.20, 99%CI (1.14, 1.25) | <0.0001 | 24% |
|  |  |  | CT | | SXT+CT |  |  | RD=0.15, 99%CI (0.11, 0.19) | <0.0001 | 8% |
|  | CSS | | CT | | SXT+CT | 12 | 1125 | WMD=-4.87, 99%CI (-6.61, -3.13) | <0.00001 | 89% |
|  | AE | Mild subcutaneous ecchymosis, nausea and abdominal bloating |  | |  |  |  |  |  |  |
| Wu et al.  (2010) | Efficacy rate | | Other injections+CT | | SXT+CT | 28 | 2718 | OR=3.30, 95%CI (2.80, 3.89) | <0.00001 | P=0.99 |
|  | CSS | | Troxerutin/ Ligustrazine/ LMWH calcium/ Compound Danshen/ Danshen/ Xueshuantong+CT | | SXT+CT | 13 | 1337 | WMD=-3.10, 95%CI (-4.33, -1.86) | <0.00001 | P<0.00001 |
| Su et al.  (2010) | Recovery rate (By ESS) | | CT | | SXT+Edaravone+CT | 6 | 447 | RR=1.75, 95%CI (1.30, 2.35) | 0.0002 | 0% |
|  |  |  | Edaravone+CT | | SXT+Edaravone+CT | 4 | 466 | RR=1.43, 95%CI (1.03, 1.97) | 0.03 | 0% |
|  | Total effective rate | | CT | | SXT+Edaravone+CT | 6 | 447 | RR=1.38, 95%CI (1.22, 1.55) | <0.00001 | 0% |
|  |  |  | Edaravone+CT | | SXT+Edaravone+CT | 4 | 466 | RR=1.16, 95%CI (1.06, 1.27) | 0.001 | 16% |
| Li and Miao  (2007) | Total effective rate | | CT | | SXT+CT | 3 | 315 | RR=1.13, 99%CI (0.98, 1.30) | 0.002 | 0% |
|  | CSS | | CT | | SXT+CT | 3 | 315 | WMD=-5.20, 99%CI (-7.89, -2.51) | <0.00001 | 93% |
|  | BI | | CT | | SXT+CT | 1 | 78 | WMD=19.40, 99%CI (17.17, 21.63) | <0.00001 | |
|  | AE | Mild subcutaneous ecchymosis |  | |  | 1 | 3 |  | | |
| Liu et al.  (2006) | Total effective rate | | Danshen+CT | | SXT+CT | 3 | 310 | OR=4.01, 95%CI (2.00, 8.04) | <0.0001 | 0% |
|  |  |  | Ligustrazine+CT | | SXT+CT | 2 | 203 | OR=4.20, 95%CI (1.48, 11.91) | 0.007 | 0% |
|  |  |  | Xuesaitong+CT | | SXT+CT | 1 | 152 | OR=3.91, 95%CI (1.03, 14.01) | 0.05 |  |
|  |  |  | Notoginseng+CT | | SXT+CT | 1 | 87 | OR=3.58, 95%CI (0.68, 18.85) | 0.13 |  |
|  |  |  | LMWH calcium+CT | | SXT+CT | 1 | 63 | OR=1.04, 95%CI (0.19, 5.57) | 0.97 |  |
|  |  |  | Troxerutin+CT | | SXT+CT | 1 | 115 | OR=8.60, 95%CI (1.02, 72.38) | 0.05 |  |
|  |  |  | Xueshuantong+CT | | SXT+CT | 1 | 100 | OR=1.74, 95%CI (0.39, 7.71) | 0.47 |  |
|  |  |  | Safflower+CT | | SXT+CT | 1 | 92 | OR=9.00, 95%CI (1.09, 74.30) | 0.04 |  |
|  |  |  | Other injections+CT | | SXT+CT | 11 | 1122 | OR=3.76, 95%CI (2.44, 5.79) | <0.00001 | 0% |
|  | LSV | | Notoginseng+CT | | SXT+CT | 1 | 87 | WMD=-2.30, 95%CI (-3.59, -1.01) | 0.0005 |  |
|  |  |  | Ligustrazine+CT | | SXT+CT | 1 | 105 | WMD=-1.15, 95%CI (-1.75, -0.55) | 0.0002 |  |
|  |  |  | Danshen+CT | | SXT+CT | 1 | 120 | WMD=-0.76, 95%CI (-0.97, -0.55) | <0.00001 | |
|  | HSV | | Notoginseng+CT | | SXT+CT | 1 | 87 | WMD=-0.53, 95%CI (-0.98, -0.08) | 0.02 |  |
|  |  |  | Ligustrazine+CT | | SXT+CT | 1 | 105 | WMD=-0.80, 95%CI (-1.08, -0.52) | <0.00001 | |
|  |  |  | Safflower+CT | | SXT+CT | 1 | 92 | WMD=0.20, 95%CI (-0.02, 0.42) | 0.08 |  |
|  |  |  | Danshen+CT | | SXT+CT | 1 | 120 | WMD=-0.46, 95%CI (-0.65, -0.27) | <0.00001 | |
|  | PV | | Notoginseng+CT | | SXT+CT | 1 | 87 | WMD=-0.13, 95%CI (-0.47, 0.21) | 0.46 |  |
|  |  |  | Ligustrazine+CT | | SXT+CT | 1 | 105 | WMD=-0.31, 95%CI (-0.43, -0.19) | <0.00001 | |
|  |  |  | Safflower+CT | | SXT+CT | 1 | 92 | WMD=-0.10, 95%CI (-0.31, 0.11) | 0.36 |  |
|  |  |  | Xueshuantong+CT | | SXT+CT | 1 | 100 | WMD=-0.38, 95%CI (-0.54, -0.22) | <0.00001 | |
|  |  |  | Danshen+CT | | SXT+CT | 1 | 120 | WMD=-0.23, 95%CI (-0.35, -0.11) | <0.00001 | |
|  | Packed cell volume | | Notoginseng+CT | | SXT+CT | 1 | 87 | WMD=-1.63, 95%CI (-3.71, 0.45) | 0.12 |  |
|  |  |  | Ligustrazine+CT | | SXT+CT | 1 | 105 | WMD=-2.29, 95%CI (-4.08, -0.50) | 0.01 |  |
|  |  |  | Xueshuantong+CT | | SXT+CT | 1 | 100 | WMD=1.72, 95%CI (0.37, 3.07) | 0.01 |  |
|  | FIB | | Notoginseng+CT | | SXT+CT | 1 | 87 | WMD=-0.50, 95%CI (-0.80, -0.20) | 0.001 |  |
|  |  |  | Ligustrazine+CT | | SXT+CT | 1 | 105 | WMD=-1.49, 95%CI (-1.72, -1.26) | <0.00001 | |
|  |  |  | Xueshuantong+CT | | SXT+CT | 1 | 100 | WMD=-0.53, 95%CI (-0.72, -0.34) | <0.00001 | |
|  |  |  | Danshen+CT | | SXT+CT | 1 | 120 | WMD=-0.43, 95%CI (-0.62, -0.24) | <0.0001 |  |
|  | EAI | | Ligustrazine+CT | | SXT+CT | 1 | 105 | WMD=-0.61, 95%CI (-0.80, -0.42) | <0.00001 | |
|  | Platelet adhesion rate | | Safflower+CT | | SXT+CT | 1 | 92 | WMD=-4.00, 95%CI (-6.46, -1.54) | 0.001 | |
|  | CSS | | LMWH calcium+CT | | SXT+CT | 1 | 63 | WMD=-0.24, 95%CI (-0.76, 0.28) | 0.36 |  |
|  |  |  | Troxerutin+CT | | SXT+CT | 1 | 115 | WMD=0.86, 95%CI (-0.76, 0.28) | 0.07 |  |
|  |  |  | Safflower+CT | | SXT+CT | 1 | 92 | WMD=-2.60, 95%CI (-3.23, -1.97) | <0.00001 | |
|  | TNF-α | | Troxerutin+CT | | SXT+CT | 1 | 115 | WMD=-0.31, 95%CI (-0.44, -0.18) | Statistically significant difference | |
|  | sICAM-1 | | Troxerutin+CT | | SXT+CT | 1 | 115 | WMD=-12.68, 95%CI (-24.95, -0.41) | Statistically significant difference | |
|  | TG | | Xuesaitong+CT | | SXT+CT | 1 | 152 | WMD=1.38, 95%CI (1.31, 1.45) | Statistically significant difference | |
|  | TC | | Xuesaitong+CT | | SXT+CT | 1 | 152 | WMD=1.10, 95%CI (1.01, 1.19) | Statistically significant difference | |
|  | HDL-C | | Xuesaitong+CT | | SXT+CT | 1 | 152 | WMD=-0.34, 95%CI (-0.38, -0.30) | Statistically significant difference | |
|  | LDL-C | | Xuesaitong+CT | | SXT+CT | 1 | 152 | WMD=0.82, 95%CI (0.44, 1.20) | Statistically significant difference | |
|  | AE | Low-grade fever resolving spontaneously within days | Xueshuantong+CT | |  | 1 | 2 |  |  |  |
